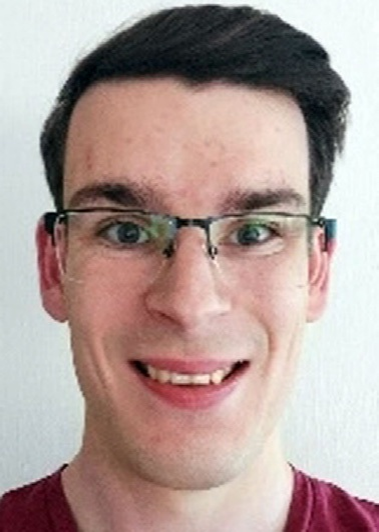# f‐Block Phospholyl and Arsolyl Chemistry

**DOI:** 10.1002/chem.202005231

**Published:** 2021-02-24

**Authors:** David P. Mills, Peter Evans

**Affiliations:** ^1^ Department of Chemistry The University of Manchester Oxford Road Manchester M139PL UK

**Keywords:** actinides, arsolyl, lanthanides, phospholyl, rare earths

## Abstract

The f‐block chemistry of phospholyl and arsolyl ligands, heavier p‐block analogues of substituted cyclopentadienyls (Cp^R^, C_5_R_5_) where one or more CR groups are replaced by P or As atoms, is less developed than for lighter isoelectronic C_5_R_5_ rings. Heterocyclopentadienyl complexes can exhibit properties that complement and contrast with Cp^R^ chemistry. Given that there has been renewed interest in phospholyl and arsolyl f‐block chemistry in the last two decades, coinciding with a renaissance in f‐block solution chemistry, a review of this field is timely. Here, the syntheses of all structurally characterised examples of lanthanide and actinide phospholyl and arsolyl complexes to date are covered, including benzannulated derivatives, and together with group 3 complexes for completeness. The physicochemical properties of these complexes are reviewed, with the intention of motivating further research in this field.

## Graphical abstract

**Rare earth and actinide** phospholyl and arsolyl chemistry is reviewed herein, focusing on structurally characterised complexes where the binding mode of the ligand is well‐defined. A range of binding modes is shown for unsubstituted phospholyl rings, where M=rare earth or actinide metal and E=P, As or Sb; arsolyl binding modes are analogous.

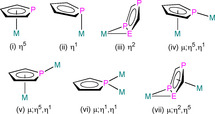

## 1 Introduction

The f‐block elements, the lanthanides (Ln) and actinides (An), exhibit remarkable physicochemical properties that have spurred numerous curiosity‐driven investigations and technological applications.^[1]^ Organometallic f‐block chemistry is predominated by cyclopentadienyl ligands (Cp, C_5_H_5_) and their derivatives (Cp^R^, C_5_R_5_), where one or more of the ring H atoms are substituted by a wide variety of alkyl, aryl or heteroatomic R groups; much of their success owes to: (i) straightforward ligand synthesis and installation at metals by well‐developed and robust synthetic routes; (ii) occupation of the equivalent of three coordination sites at large f‐block ions in their most common η^5^‐binding mode; and, (iii) facile tuning of ligand steric and electronic properties by R group variation to provide additional kinetic and thermodynamic stabilisation and fine‐control of metal coordination spheres and redox chemistry.^[2]^ Cp and Cp^R^ ligands have supported seminal examples of f‐block chemistry in both a spectator ligand role and in controlling the physicochemical properties, including rare examples of f‐block‐metal(loid) bonds^[3]^ and terminal unsupported multiple bonds between f‐block and p‐block elements,^[4]^ rich single‐electron transfer (SET) chemistry,^[5]^ the discovery of hitherto unknown +2 oxidation states in solution for a wide range of Ln and An,^[6]^ and Ln single‐molecule magnets (SMMs) with high blocking temperatures.^[7]^

Given the huge influence of substituents in f‐block Cp^R^ chemistry, the comparative dearth of examples of isoelectronic heterocyclopentadienyl f‐block complexes, where one or more of the ring C atoms is substituted by other p‐block atoms, is noteworthy.^[8]^ Of these related ligand families, phospholyls (C_5−*n*_R_5−*n*_P_*n*_) and arsolyls (C_5−*n*_R_5−*n*_As_*n*_) have proved popular, with the lighter congeners more widely investigated, and their group 3 and f‐block metal chemistry was reviewed several times between 1998 and 2006.[^3a^, ^8^, ^9^] The relatively restricted development of f‐block phospholyl and arsolyl chemistry compared with that of Cp and Cp^R^ analogues is mirrored in the s‐, p‐ and d‐blocks.[^9a^, ^9b^] The reasons for this disparity are the same as for other heterocyclopentadienyls: the well‐documented benefits of Cp and Cp^R^ ligands summarised above, together with their widespread renown, make them natural primary choices for chemists in exploratory synthesis fields.[^2a^, ^2b^] However, for more nuanced and specific applications, the introduction of ring heteroatoms can provide electronic fine‐tuning to maximise physicochemical properties, providing rich and diverse chemistry.^[8]^

The relative popularity of phospholyls and arsolyls in f‐block chemistry compared with other heterocyclopentadienyls can be attributed to both pragmatic (i–ii) and ligand design (iii–vi) considerations:[^3^, ^8^, ^9^] (i) synthetic routes to monophospholyls and ‐arsolyls are mature and are relatively straightforward; (ii) ^31^P nuclei are *I*=1/2 with 100 % natural abundance (^75^As *I*=3/2, 100 % abundant), providing a useful NMR/EPR spectroscopic handle; (iii) phospholyl and arsolyl ligands are relatively soft compared with Cp^R^ analogues and are thus well‐suited for stabilising low oxidation state f‐block ions; (iv) phospholyls and arsolyls are able to bind in a η^1^‐fashion through their P/As lone pairs but are more likely to exhibit an η^5^‐binding mode than lighter congeners with harder heteroatom donor atoms, for example, pyrrolyl (C_4_R_4_N) and pyrazolyl/imidazolyl (C_3_R_3_N_2_), thus they more effectively mimic Cp^R^ ligands in occupying a large proportion of metal coordination spheres; (v) the P and As lone pairs provide a range of alternative binding modes over Cp^R^; for example, for monophospholyls μ:η^5^,η^1^‐ and μ:η^1^‐binding modes increase the likelihood of formation of multinuclear complexes; and, (vi) phospholyls and arsolyls are poorer π‐donors and stronger π‐acceptors than analogous Cp^R^ ligands, influencing metal reduction potentials and redox chemistry.

Since the first rare earth phospholyl complexes were reported by Nief and Mathey in 1989,^[10]^ <100 monophospholyl, monoarsolyl and polyphospholyl complexes of the group 3 metals, Ln and An (including benzannulated derivatives) have been structurally authenticated to date; this contrasts starkly with the corresponding Cp/Cp^R^ chemistry, where the first reported examples were by Birmingham and Wilkinson in 1954^[11]^ and there are now thousands of structurally characterised complexes.^[12]^ As noted above, various books and reviews have covered group 3 and f‐block metal phospholyl and arsolyl chemistry prior to 2006 as part of wider subject areas.[^3a^, ^8^, ^9^] In the last fifteen years, there have been significant discoveries that we believe now warrant a review solely dedicated to this topic.

Here, we will firstly present an overview of the ligand design criteria and binding modes of phospholyls and arsolyls, followed by general synthetic routes to these ligands and metal complexes; we focus on selected examples for brevity as this material is covered in detail elsewhere.[^3a^, ^8^, ^9^] We then review all structurally authenticated group 3 and f‐block monophospholyl and ‐arsolyl complexes, divided into separate sections for Ln and An, and subdivided by formal metal oxidation state; in the case of the most developed Ln^III^ chemistry this is further split by Ln starting materials and ancillary ligands. The small number of examples of polyphospholyl Ln and An complexes are covered together at the end in a dedicated section, subdivided by the number of P atoms in the rings. As we focus on structurally characterised examples, we compile these complexes and salient data at the end (Table 1) and we only provide ligand binding modes where these have been authenticated in the solid state. When appropriate, we cover interesting physicochemical properties that the complexes have been shown to exhibit, within individual sections. We conclude with remarks on the current and predicted future state of group 3 and f‐block phospholyl and arsolyl chemistry. We will include the group 3 elements Sc, Y and La under the heading of Ln in this review, as they can be considered as diamagnetic M^III^ mimics of Ln^III^ ions, although we appreciate the term “rare earth” is the preferred nomenclature for the group 3 and Ln metals combined.^[1a]^

**Table 1:** Structurally characterised Ln and An phospholyl and arsolyl complexes covered in this review, with range of M−P distances and ^31^P NMR spectroscopy data where available.

Complex	Molecular formula	Range M−P/As [Å]	*δ*_P_ [ppm]	Reference
*5.1. Ln* ^*II*^ *monophospholyl and ‐arsolyl complexes*				
**1** **2** **3** **4** **5** **6** **7** **8** **9** **10** **11** **12** **13** **14** **15‐Sm** **15‐Yb** **16** **17** **18**	[Yb(η^5^‐Dpp)_2_(THF)_2_] [Sm(η^5^‐Bdmp)_2_(THF)_2_] [Sm(η^1^‐Dbp)_2_(THF)_4_] [{Yb(η^5^‐Tmp)(μ‐Cl)(THF)_2_}_2_] [{Yb(η^5^‐Tmp)(μ‐SPh)(THF)_2_}_2_] [{Yb(THF)_2_(μ;η^5^,η^1^‐Tmp)_2_}Ru(H)_2_(PPh_3_)_2_] [Tm(Dtp)_2_(THF)] [Tm(Dsas)_2_(THF)] [{Sm(η^5^‐Dtp)(μ:η^5^,η^1^‐Dtp)}_2_] [{Sm(η^5^‐Dsp)(μ:η^5^,η^1^‐Dsp)}_2_] [Tm(Dtp)_2_] [Tm(Htp)_2_(THF)] [Tm(Hsp)_2_(THF)] [{(η^5^‐Htp)Tm(μ:η^5^,η^1^‐Htp)}_2_] [Sm(η^5^‐Tmp)_2_(py)_2_] [Yb(η^5^‐Tmp)_2_(py)_2_] [Yb(η^5^‐Tmp)(η^1^‐Tmp)(L^Et^)] [Yb(η^5^‐Tmp)(η^1^‐Tmp)(L^Cy^)] [Yb(η^5^‐Tmp)_2_(L^Ph^)]	2.959(1)–2.986(1) 3.0775(1) 3.1908(6) 2.911(1) 2.931(4)–2.955(5) 2.930(2) 2.943(1)–2.967(1) 2.968(1)–2.9759(8) 3.045(1)–3.197(1) 3.023(1)–3.168(1) 2.867(2)–2.875(2) 2.941(2)^[b]^ 2.921(1)^[b]^ 2.954(2)–3.028(2) 3.043(1)–3.046(1) 2.903(8)–2.941(2) 2.9723(8)–3.027(1) 2.925(1)–3.023(1) 2.947(1)–2.9480(8)	74.7 – – 81.4 82.5 103 −338.3 −265.7 −519^[a]^ −383^[a]^ −257 −290 −235 −290^[a]^ −624 79.5 77.9 79.8 79.0	[18] [20] [20] [21] [21] [22] [23] [23] [25] [25] [25] [26] [26] [27] [28] [29] [29] [29] [29]
5.2. Ln^III^ monophospholyl and ‐arsolyl complexes				
**19** **20** **21** **22‐P** **22‐As** **23** **24** **25** **26** **27** **28** **29** **30** **31** **32** **33** **34** **35** **36** **37‐Dy** **37‐Tm** **38** **39** **40** **41** **42** **43‐La** **43‐Ce** **43‐Nd** **43‐Sm** **44‐La** **44‐Ce** **45** **46** **47‐Sc** **47‐Y** **47‐Sm** **48** **49** **50** **51** **52** **53** **54‐Y** **54‐Tb** **54‐Dy** **54‐Er** **54‐Tm**	[Sm(η^5^‐Tmp)_2_(O*t*Bu)(THF)] [Sm(Cp*)_2_(η^5^‐Htp)] [Sm(Cp*)_2_(η^1^‐Tmp)] [Sm(Cp*)_2_(μ:η^5^,η^1^‐Mhp)Sm(Cp*)_2_(η^1^‐Mhp)] [Sm(Cp*)_2_(μ:η^5^,η^1^‐Mhas)Sm(Cp*)_2_(η^1^‐Mhas)] [{Sm(Cp*)_2_(μ:η^5^,η^1^‐C_4_H_4_P)}_2_] [Sm(η^5^‐Dsp)_2_(N_2_Ph_2_)] [Tm(η^5^‐Dtp)_2_(N_2_Ph_2_)] [{Tm(η^5^‐Dtp)_2_}_2_(μ‐S)] [{Sm(η^5^‐Tmp)_2_}_2_(μ‐NC_13_H_9_‐C_13_H_9_N)] [Tm(η^5^‐Dtp)_2_(tmbp)] [Tm(η^5^‐Dtp)_2_(bipy)] [Tm(η^5^‐Dtp)(bipy)_2_] [Tm(η^5^‐Dtp)_2_(η^1^‐Dtp)] [{Sm(η^5^‐Tmp)(μ:η^5^,η^1^‐Tmp)_2_(μ‐Cl)K(C_7_H_8_)}_2_]_∞_ [{Sm(η^5^‐Mhp)_2_(μ:η^5^,η^1^‐Mhp)}_2_] [Sm(η^5^‐Tmp)(μ:η^5^,η^1^‐Tmp)(μ^3^‐Cl)_2K(_Et_2_O)]_∞_ [Sc(η^5^‐Tmp)_2_(μ‐Cl)_2_Li(TMEDA)] [{Sc(η^5^‐Dtp)(μ‐Cl)(Cl)(py)}_2_] [Dy(η^5^‐Dtp)_2_(I)] [Tm(η^5^‐Dtp)_2_(I)] [{Tm(η^5^‐Htp)_2_(μ‐I)}_2_] [{Dy(η^5^‐Dsp)_2_(μ‐I)}_2_] [Sm(η^5^‐Dtp)(I)_2_(THF)_2_] [Dy(η^5^‐Dtp)_2_][Al{OC(CF_3_)_3_}_4_] [K(18‐crown‐6)(THF)_2_][Nd(η^5^‐Tmp)_2_(BH_4_)_2_] [{La(η^5^‐Htp)_2_(μ‐BH_4_)}_2_] [{Ce(η^5^‐Htp)_2_(μ‐BH_4_)}_2_] [{Nd(η^5^‐Htp)_2_(μ‐BH_4_)}_2_] [{Sm(η^5^‐Htp)_2_(μ‐BH_4_)}_2_] [{La(η^5^‐Htp)_2_(μ‐BH_4_)_2K(_μ‐DME)_2_}_2_] [{Ce(η^5^‐Htp)_2_(μ‐BH_4_)_2K(_μ‐DME)_2_}_2_] [{Ce(η^5^‐Htp)_2_(BH_4_)_2K(_OEt_2_)(THF)]_∞_ [Sc(η^5^‐Tmp){CH(SiMe_3_)_2_}(μ‐Cl)_2_Li(TMEDA)] [Sc(η^5^‐Dtp)(κ^2^‐CH_2_C_6_H_4_NMe_2_‐*o*)_2_] [Y(η^5^‐Dtp)(κ^2^‐CH_2_C_6_H_4_NMe_2_‐*o*)_2_] [Sm(η^5^‐Dtp)(κ^2^‐CH_2_C_6_H_4_NMe_2_‐*o*)_2_] [{La(μ:η^5^,η^1^‐Tmp)(AlMe_4_)_2_}_2_] [Nd(η^5^‐Tmp)(AlMe_4_)_2_] [Nd(η^5^‐Dsp)(AlMe_4_)_2_] [Nd(η^5^‐Tmp)(AlMe_4_){OSi(O*t*Bu)_3_(AlMe_3_)}] [Nd(COT)(η^5^‐Tmp)(HMPA)] [Nd(COT)(η^5^‐Dsp)(THF)] [Y(COT)(η^5^‐Dsp)] [Tb(COT)(η^5^‐Dsp)] [Dy(COT)(η^5^‐Dsp)] [Er(COT)(η^5^‐Dsp)] [Tm(COT)(η^5^‐Dsp)]	2.951(2)–3.026(2) 3.153(1) 2.856(1)–2.891(1) 2.886(1)–3.1032(8) 2.9776(8)–3.1610(6) 3.101(2)–3.274(1) 2.9484(6) 2.869(1) 2.875(7)^[b]^ 2.889(5)–2.937(5) 2.825(2)–2.862(2) 2.843(2)–2.844(2) 2.841(2) 2.8135(8)–2.8727(7) 2.924(4)–2.953(3) 2.9270(5)–2.9978(5) 2.905(2)–2.926(1) 2.694(2)–2.718(2) 2.6960(5) 2.9235(2) 2.8119(12)–2.8167(12) 2.906(2)–2.9504(14) 2.8500(7)–2.8690(7) 2.9112(14) 2.7880(8)–2.7981(8) 2.982(3)–2.995(3) 3.089(5)–3.138(3) 3.058(5)–3.099(4) 3.019(11)–3.077(6) 3.016(6)–3.054(4) 3.1790(7)–3.1869(7) 3.1456(13)–3.1534(13) 3.1488(13)–3.1682(12) 2.712(2) 2.769(1) 2.928(1) 3.009(1) 3.0604(3)–3.1962(3) 2.9252(1) 2.8972(3) 2.9652(6) 2.968(8) 3.1095(4) 2.8261(6) 2.8745(12) 2.8577(13) 2.7929(11) 2.7823(12)	– – – – – – 148 – – 43.24, 46.75 – – – – –^[c]^ 9.3^[a]^ 49.7 99.8 123.0 – – – – 77.5 – – 105.65 – – – 96.49 – – 119.2 99.0 88.9 62.9 128.4 444.0 484.1 544 – – 157.96 – – – –	[30] [31] [31] [31] [31] [31] [25] [25] [25] [28] [32] [32] [32] [33] [34] [34] [35] [36] [37] [38] [27] [27] [38] [37] [39] [41] [42] [42] [42] [42] [42] [42] [42] [36] [37] [37] [37] [43] [43] [43] [44] [46] [47] [48] [48] [48] [48] [48]
6.1. An^III^ monophospholyl and ‐arsolyl complexes				
**55** **56‐P** **56‐As**	[U(η^5^‐Tmp)(μ:η^5^,η^1^‐Tmp)(BH_4_)]_2_ [U{C_8_H_6_(Si*i*Pr_3_)_2_‐1,4}(η^5^‐Tmp)(THF)] [U{C_8_H_6_(Si*i*Pr_3_)_2_‐1,4}(η^5^‐Tmas)(THF)]	2.945(3)–2.996(3) 2.776(15)–2.9868(14) 3.0781(7)	727, 3471 846.2 –	[51] [52] [52]
6.2. An^IV^ monophospholyl complexes				
**57** **58** **59** **60** **61** **62**	[U(η^5^‐Tmp)_2_(BH_4_)_2_] [U(η^5^‐Tmp)_3_(Cl)] [U(η^5^‐Tmp)(Cl)_3_(DME)] [U(COT)(η^5^‐Tmp)(BH_4_)(THF)] [{U(Cl)_2_(μ;η^5^,η^1^‐Tmp)_2_}Ni(μ;η^1^,η^1^‐Tmp)]_2_ [U(Cl)_2_(μ:η^5^,η^1^‐Tmp)_2_Ni(μ:η^1^‐Tmp)_2_Ni(μ:η^5^,η^1^‐Tmp)_2_U(Cl)_2_]	2.905(1) 2.927(4) 2.926(4) 2.970(8) 2.823(7)–2.862(7) 2.851(9)–2.86(1)	960 – – – 199.2 –	[49] [53] [54] [55] [56] [56]
7.1. Ln and An C_3_P_2_ and C_2_P_3_ complexes				
**63** **64‐E** **65‐E** **66‐Sc** **66‐Y** **66‐Tm** **66‐U** **67** **68**	[Sc{η^5^‐C_2_ *t*Bu_2_P_3_)}_2_(μ:η^6^,η^6^‐C_3_ *t*Bu_3_P_3_)] [Sm(Cp*)_2_(η^2^‐C_2_ *t*Bu_2_P_2_E)(THF)] (E=P, Sb) [Li(THF)_4_][Yb(η^5^‐C_2_ *t*Bu_2_P_2_E)_2_(η^2^‐C_2_ *t*Bu_2_P_2_E)] (E=P, Sb) [Sc(η^5^‐C_2_ *t*Bu_2_P_3_)_2_(η^2^‐C_2_ *t*Bu_2_P_3_)] [Y(η^5^‐C_2_ *t*Bu_2_P_3_)_2_(η^2^‐C_2_ *t*Bu_2_P_3_)] [Tm(η^5^‐C_2_ *t*Bu_2_P_3_)_2_(η^2^‐C_2_ *t*Bu_2_P_3_)] [U(^η5^‐C_2_ *t*Bu_2_P_3_)_2_(η^2^‐C_2_ *t*Bu_2_P_3_)] [(η^5^‐C_2_ *t*Bu_2_P_3_)_2_Sc(μ:η^2^,η^5^‐C_2_ *t*Bu_2_P_3_)Sc(η^5^‐C_2_ *t*Bu_2_P_3_)] [{Eu(diglyme)_2_(μ‐CCPh)}_2_][C_3_ *t*Bu_3_P_2_]_2_	2.802(2)–2.877(2) 3.135(2)–3.164(2) 3.09(2)^[d]^ 2.762(3)–2.813(3) 2.884(2)–3.059(3) 2.853(2)–3.052(3) 2.968(2)–3.114(2) 2.5627(14)–2.942(2) –	– –^[c]^ –^[c]^ 265.0, 296.5 263.9, 289.9 – 691.5 – –	[57] [62] [62] [63] [64] [64] [64] [64] [65]
7.2. Ln and An planar cyclo‐P_5_ complexes				
**69** **70**	[{(Sm(Cp*)_2_}_3_(μ:η^1^,η^1^,η^2^,η^2^‐*cyclo*‐P_5_){Mo(Cp)(CO)_2_}_3_] [{U[N(CH_2_CH_2_NSi*i*Pr_3_)_3_]}_2_(μ:η^5^,η^5^‐*cyclo*‐P_5_)]	2.978(11)^[d]^ 3.250(6)–3.335(6)	– –	[67] [68]

[a] Value for THF‐coordinated monomer. [b] Mean value. [c] Various species in solution. [d] Unreliable metrical parameters owing to crystallographic disorder.

## 2 Ligand Design Criteria and Binding Modes

**Figure 1:**
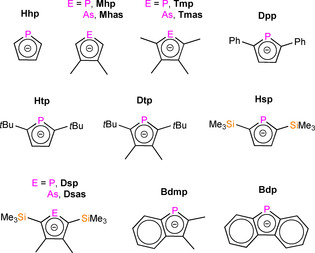
Monophospholyl and arsolyl ligands in f‐block chemistry, with acronyms used in this review that are commonplace in the literature.

**Figure 2:**
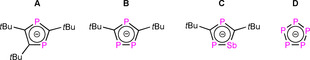
Polyphospholyl ligands in f‐block chemistry, labelled **A**–**D**.

The phospholyl and arsolyl ligands that have been employed in f‐block chemistry to date are compiled in Figures 1 and 2; acronyms are provided for monophosphoyls and ‐arsolyls, whereas polyphospholyls are labelled **A**–**D**. Monophospholyl and ‐arsolyl ligands are variously substituted at the 2,5‐, 3,4‐, or all four C‐positions of the C_4_E (E=P, As) rings, apart from the parent phospholyl C_4_H_4_P, Hhp. Substituents include R=Me, *t*Bu, SiMe_3_ and Ph, and benzannulated derivatives; the currently available selection and ring positions are intrinsically linked to the common synthetic routes to these ligands, as can be deduced from only *t*Bu substituents being seen in the polyphospholyls **A**–**C** (see Section 3). As stated previously, the introduction of P or As into carbocyclic rings influences both the strength of metal–ligand binding and the resultant redox properties of complexes.[^3a^, ^8^, ^9^] As with Cp^R^ chemistry,^[2]^ the substituents affect complex solubility and both the thermodynamic and kinetic stability of f‐block complexes; the electron density of the rings is also influenced by donating (Me, *t*Bu) and withdrawing (SiMe_3_, Ph, fused carbocyclic rings) R groups. Ligands with the largest R groups tend to give the most kinetically stable complexes, which are less likely to oligomerise owing to a combination of steric bulk about the metal coordination sphere and buttressing of the heteroatom lone pairs.

**Figure 3:**
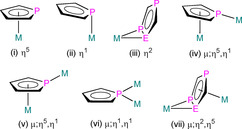
Common binding modes (i)–(vii) of mono‐ and polyphospholyl ligands, shown for unsubstituted rings, where M=metal and E=P, As or Sb; arsolyl binding modes are analogous.

The most common binding modes of mono‐ and polyphospholyls are compiled in Figure 3, with analogous hapticities seen for monoarsolyls. The introduction of heteroatoms with lone pairs increases the flexibility of ligand coordination over the most common η^5^‐, η^3^‐ and η^1^‐binding of Cp^R^ ligands, where bridging modes are rare for the f‐block.^[2]^ As stated previously, the η^5^‐binding mode is the most common binding mode for phospholyls and arsolyls with f‐block elements as P and As atoms are relatively soft. Substituents, available space at metal coordination spheres and ancillary ligands are all contributory factors as to whether or not the heteroatom lone pairs form dative bonds with f‐block ions.[^3a^, ^8^, ^9^] Although this review focuses on binding modes observed in solid‐state structures, it must be appreciated that dynamic fluxional behaviour in solution is common, and the presence of ^31^P or ^75^As nuclei can provide an additional useful handle to study this behaviour by NMR or EPR spectroscopy.

## 3 Synthetic Routes to Phospholyls and Arsolyls

**Scheme 1:**
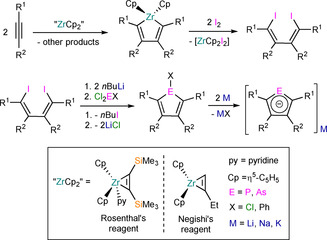
Synthesis of alkali metal monophospholyl and ‐arsolyl complexes (inset shows key).

Monophospholyl and ‐arsolyl pro‐ligands are typically prepared from the corresponding alkyne starting materials (R^1^C≡CR^2^) by the synthetic routes outlined in Scheme 1, or by variations of these methods.[^3a^, ^8^, ^9^] Alkynes are first reductively coupled with a zirconocene species “ZrCp_2_” to generate the corresponding metallacycles [Zr(Cp)_2_(C_4_R^1^_2_‐2,5‐R^2^_2_‐3,4)], in which the less sterically demanding substitutes are selectively placed in the β‐positions (R^1^>R^2^ with respect to steric bulk). The zirconocene species is typically generated in situ, historically from Negishi's reagent “[Zr(Cp)_2_(η^2^‐CH_2_CHCH_2_CH_3_)]”; however, Rosenthal's reagent [Zr(Cp)_2_(η^2^‐Me_3_SiCCSiMe_3_)(py)] (py=pyridine) offers multiple advantages over species generated in situ, such as: significantly greater thermal stability in the solid state, solubility in non‐donor solvents and generally provides higher yields of the targeted metallacycle.^[13]^ Alternatively, low oxidation state titanium reagents, such as [Ti(O*i*Pr)_2_(η^2^‐propene)], may be used as reductive coupling reagents but these tend to exhibit lower functional group tolerances than zirconocene‐based reagents.^[14]^

The zirconium metallacycles [Zr(Cp)_2_(C_4_R^1^_2_‐2,5‐R^2^_2_‐3,4)] are treated with diiodine to liberate [Zr(Cp)_2_(I)_2_] and the respective 1,4‐diiodobutadienes. Following work‐up and recrystallisation, the dienes are then subjected to a metal–halogen exchange reaction with *n*BuLi, and the dilithio salt generated in situ is treated with Cl_2_EX (E=P, As; X=Cl, Ph) to yield the respective cyclic phenyl‐ or chloro‐substituted phosphole or arsole XE(C_4_R^1^_2_‐2,5‐R^2^_2_‐3,4) by a salt metathesis reaction. In some cases, these heterocycles may be prepared more directly by a σ‐bond metathesis reaction of the zirconium metallacycles [Zr(Cp)_2_(C_4_R^1^_2_‐2,5‐R^2^_2_‐3,4)] with parent ECl_3_ (E=P, As). The P/As−X bond in the substituted phosphole or arsole is cleaved with at least two equivalents of an alkali metal to generate the corresponding alkali metal monophospholyl or ‐arsolyl salts [M(EC_4_R^1^_2_‐2,5‐R^2^_2_‐3,4)] (M=Li, Na, K), which are used as ligand transfer reagents to generate f‐element complexes.

**Scheme 2:**
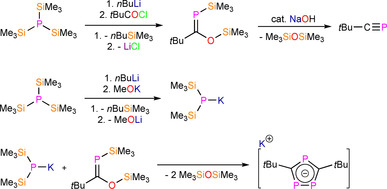
Exemplar synthetic route to polyphospholyl **B**; *t*BuC≡P is also a common precursor to **A**–**C** (see Section 7).

Owing to the synthetic ease of functionalising alkynes, the relatively low cost of starting materials, and the high functional group tolerance of the alkyne coupling reactions, a diverse range of monophospholyl and ‐arsolyl ligands can be readily obtained and have been installed at f‐block metals (Figure 1). There are significantly fewer reported examples of f‐block complexes featuring polyphospholyl ligands **A**–**D** (Figure 2).[^3a^, ^8^, ^9^] This can be attributed to **A**–**C** being prepared by multistep syntheses with hazardous reagents such as P(SiMe_3_)_3_ and *t*BuCP (e.g., Scheme 2). The synthesis of P(SiMe_3_)_3_ from red phosphorus, sodium and Me_3_SiCl can be disconcerting, and this is prohibitively expensive to purchase in bulk; removing the reliance on *t*BuCP could thus facilitate the rapid development of polyphospholyl chemistry. The *cyclo*‐P_5_ ring (**D**) is typically assembled directly at the metal from white phosphorus, which also presents significant hazards.^[15]^

## 4 Synthetic Routes to Complexes

A number of synthetic strategies have been developed for the synthesis of f‐block phospholyl, arsolyl and polyphospholyl complexes, with the route depending upon the nature of both the metal and ligand employed, as well as the metal oxidation state.[^3a^, ^8^, ^9^] The four most common strategies to monophospholyls and ‐arsolyls and practical considerations will be briefly outlined in this section, in decreasing order of their frequency of application; examples will be provided throughout Sections 5–6. Various synthetic routes to f‐block polyphospholyl complexes will be discussed separately with dedicated schemes in Section 7.

### 4.1 Salt metathesis

Salt metathesis reactions between Ln and An halides or pseudo‐halides with alkali metal ligand transfer agents are by far the most common route for synthesising f‐block phospholyl and arsolyl complexes.[^3a^, ^8^, ^9^] This is typically due to the commercial availability and facile synthetic routes to anhydrous and donor‐solvent coordinated trihalides for all the Ln (with the exception of PmX_3_), which can be used to prepare pseudo‐halide complexes, for example, Ln(BH_4_)_3_(THF)_3_ and Ln(AlMe_4_)_3_; some Ln diiodides are also readily available (Sm, Eu, Tm and Yb here; although DyI_2_ and NdI_2_ are known,[^5b^, ^5c^, ^5d^, ^5e^, ^5f^] they have not been utilised successfully in Ln^II^ phospholyl chemistry to date).^[1a]^ Conversely, the two An with lowest radiological hazard and highest natural abundancies, thorium and uranium, have well‐developed synthetic routes to readily solvated halide (e.g., AnCl_4_, UI_3_) and pseudo‐halide (e.g., U(BH_4_)_*n*_, *n=*3, 4) precursors from nitrate (Th), and oxide and metallic (U) starting materials.^[1b]^ Most f‐block halide and borohydride precursors can be converted to donor solvent adducts, typically THF or DME, to endow improved solubility, which facilitates their salt metathesis reactions, but the presence of these solvents can also lead to unwanted side‐products, for example, diprotonation and ring‐opening reactions of THF.

The choice of ligand transfer reagent and reaction solvent are crucial for determining the composition of products because of the highly electropositive nature of the f‐block elements.[^1^, ^16^] In the majority of cases where lithium phospholyls are used as transfer reagents salt‐occluded complexes tend to form, where Li is trapped in the coordination sphere through contacts with several Ln‐/An‐bound halides. A combination of Ln or An di‐/tri‐iodides and sodium or potassium transfer reagents often yields discrete f‐block complexes by assisting the precipitation of alkali metal iodide by‐products; potassium iodide is only sparingly soluble in THF and is therefore a desirable by‐product. Although the occlusion of such salts is suppressed with these reagents, a handful of potassium ‘ate’ f‐block phospholyl complexes have been isolated and are included in this review. The high affinity of f‐block ions for binding ethereal solvents can make it challenging to synthesise solvent‐free phospholyl and arsolyl complexes as diethyl ether or THF are typically used as the reaction solvents for solubility reasons. Whilst some metal‐bound solvent molecules can be removed from f‐block complexes upon exposure to vacuum, the elevated temperatures often required to facilitate the dissociation of strongly bound N‐ and O‐donor solvents can be greater than the temperature of complex decomposition. As a consequence, some solvent‐free f‐block phospholyl and arsolyl complexes are synthesised by reacting binary Ln or An halides with sodium or potassium ligand transfer agents in toluene at reflux for extended periods to circumvent the low solubility of the reactants in aromatic solvents.[^3a^, ^8^, ^9^] These reactions are moderate‐ to high‐yielding and have facilitated numerous studies of the resultant rare earth phospholyl complexes.

### 4.2 Redox transmetallation

Whilst salt metathesis is often the most convenient synthetic strategy for preparing f‐block phospholyl and arsolyl complexes, several alternative approaches have been developed, which in some cases can be more suitable. Redox transmetallation reactions using ligand transfer reagents of readily reducible metal ions such as Tl^I^ and Pb^II^ have proved useful for concomitant ligand installation and f‐block metal ion oxidation, in cases where the Ln or An ions have suitable redox potentials, for example, Sm^II^ and U^III^.^[1]^

### 4.3 Bond insertion

Biphospholes and biarsoles containing E−E bonds, and phospholes and arsoles containing E−X bonds (e.g., X=halide, Ph), may react directly with metallic Ln and An by a formal bond insertion with metal oxidation and ligand reduction. This has proved most useful to date for Ln^II^ phospholyl and arsolyl chemistry for Sm and Yb.[^3a^, ^8^, ^9^]

### 4.4 Redox

As phospholyl and arsolyl ligands have proven utility for stabilising metals in low oxidation states it is unsurprising that when these ligands have been installed on f‐block metals in intermediate oxidation states, the resultant complexes can often be straightforwardly oxidised or reduced, for example, Ln^II^ to Ln^III^ or U^III^ to U^IV^, and vice versa.

## 5 Lanthanide Monophospholyl and ‐arsolyl Complexes

### 5.1 Ln^II^ complexes

**Scheme 3:**
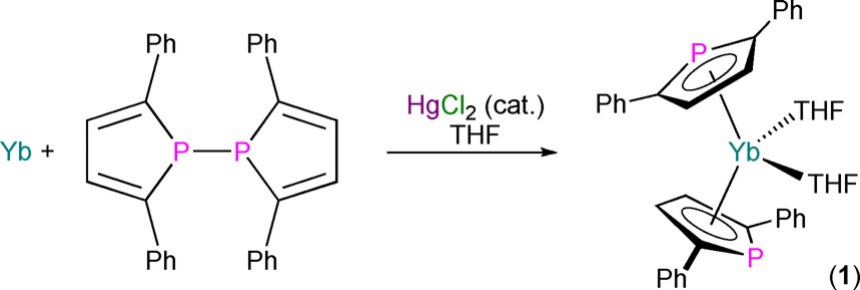
Synthesis of **1** by the oxidative insertion reaction of Yb powder with the parent biphosphole in THF in the presence of HgCl_2_.^[18]^

In 1991, Nief and Mathey communicated the synthesis of the first Ln^II^ phospholyl complexes [Ln(Tmp)_2_(THF)_2_] (Ln=Sm, Yb), by the salt metathesis reactions of the respective LnI_2_ precursor with two equivalents of K(Tmp), or the oxidative insertion reactions of Ln powders with the parent biphosphole.^[17]^ In a full paper published two years later, the corresponding bis(arsolyl) analogues [Ln(Tmas)_2_(THF)_2_] (Ln=Sm, Yb) were reported to form by analogous methods, and the related Ln^II^ complexes [Ln(η^5^‐Dpp)_2_(THF)_2_] (Ln=Sm; Yb, **1**) were prepared by adapted procedures where a trace amount of HgCl_2_ was added to promote the reactions of Ln powders with a phenyl‐functionalised biphosphole (Scheme 3).^[18]^ The Dpp‐substituted complexes did not appear to desolvate upon exposure to vacuum, but the Tmp‐ and Tmas‐substituted complexes were found to rapidly lose THF in vacuo to give donor solvent‐free variants; this provides a juxtaposition to the sluggish removal of THF from [Sm(Cp*)_2_(THF)_2_] (Cp*=C_5_Me_5_) under dynamic vacuum.^[19]^ [Ln(Tmp)_2_(THF)_2_], [Ln(Tmas)_2_(THF)_2_] and [Ln(η^5^‐Dpp)_2_(THF)_2_] were variously characterised by microanalysis and ^1^H and ^13^C NMR spectroscopy in THF, with some derivatives additionally probed by ^31^P NMR spectroscopy (*δ*_P_: [Ln(Tmp)_2_(THF)_2_], −580 ppm, Ln=Sm; 81.2 ppm *J*_YbP_=100 Hz, Ln=Yb; [Ln(η^5^‐Dpp)_2_(THF)_2_], −417 ppm, Ln=Sm; 74.7 ppm, Ln=Yb) and ^171^Yb NMR spectroscopy at −30 °C (*δ*_Yb_: [Yb(Tmp)_2_(THF)_2_], 242 ppm, *J*_YbP_=100 Hz; [Yb(Tmas)_2_(THF)_2_], 316 ppm); the Tmp rings of [Yb(Tmp)_2_(THF)_2_] were found to be fluxional at room temperature.[^17^, ^18^] The solid‐state structure of **1** was confirmed by single‐crystal X‐ray crystallography, showing a bent metallocene‐type geometry with staggered η^5^‐Tmp rings (Yb−P: 2.959(1) and 2.986(1) Å) and mutually *cis*‐THF molecules; the phenyl groups of the Dpp ligands of **1** are co‐planar with the phospholyl rings.^[18]^

**Figure 4:**
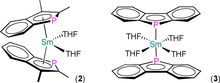
Complexes **2** and **3**.^[20]^

Nief reported the synthesis of the benzannulated Sm^II^ phospholyl complexes [Sm(η^5^‐Bdmp)_2_(THF)_2_] (**2**) and [Sm(η^1^‐Bdp)_2_(THF)_2_] (**3**; Figure 4) by standard salt metathesis from SmI_2_(THF)_2_ (**2**) or oxidative insertion (**3**) strategies in THF in 1994.^[20]^ A broad signal was observed at −694 ppm in the ^31^P NMR spectrum of **2**, and a combination of variable‐temperature NMR experiments indicated η^5^‐binding of Bdmp ligands in THF solution. This arrangement of ligands was confirmed in the solid state by single‐crystal XRD, with the structure of **2** analogous to that of **1**,^[18]^ but with a longer Ln−P distance (3.0775(1) Å)^[20]^ owing to Sm being larger than Yb.^[1a]^ The Sm−P distance of **3** is even longer at 3.1908(6) Å, owing to the mutually *trans*‐Bdp ligands adopting η^1^‐binding modes, which allow for the coordination of two additional THF molecules, leading to a distorted octahedral geometry. The authors attributed the alternative η^1^‐coordination of the Bdp ligands in **3** to the bis‐benzanulated system having reduced aromaticity about the phospholyl ring, by analysis of the degree of pyramidalisation of the P atom and intra‐ring distances.

**Figure 5:**
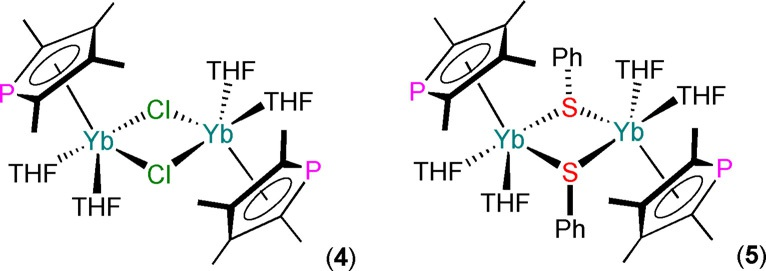
Complexes **4** and **5**.^[21]^

Also in 1994, Nief and Ricard reported the syntheses of the heteroleptic dinuclear Yb^II^ complexes [{Yb(η^5^‐Tmp)(μ‐Cl)(THF)_2_}_2_] (**4**) and [{Yb(η^5^‐Tmp)(μ‐SPh)(THF)_2_}_2_] (**5**; Figure 5) by the oxidative insertion reactions of Yb powder with the respective reagent XPC_4_Me_4_ (X=Cl or SPh) in THF with a trace amount of HgCl_2_. Complex **4** could also be prepared by the ligand scrambling reaction of [Yb(Tmp)_2_(THF)_2_] with YbCl_2_ in THF; the salt elimination reaction of **4** with two equivalents of NaSPh in THF also gave **5**.^[21]^ Complexes **4** and **5** exhibit similar geometries in the solid state, with half‐sandwich motifs at Yb with η^5^‐Tmp ligands (Yb−P: 2.911(1) Å for **4**; 2.931(4) and 2.955(5) Å for **5**), Yb_2_X_2_ cores, and each Yb coordination sphere completed by two bound THF molecules. The ^31^P NMR spectra of **4** (81.4 ppm) and **5** (82.5 ppm) each exhibited one signal, with no *J*_YbP_ coupling constants reported.

**Scheme 4:**
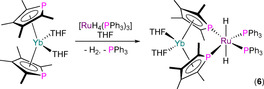
Synthesis of **6** by the reaction of [Yb(Tmp)_2_(THF)_2_] with [RuH_4_(PPh_3_)_3_].^[22]^

Desmurs et al. reported the synthesis of [{Yb(THF)_2_(μ;η^5^,η^1^‐Tmp)_2_}Ru(H)_2_(PPh_3_)_2_] (**6**) in 1996 by the coordination of [Yb(Tmp)_2_(THF)_2_] to [RuH_4_(PPh_3_)_3_], with loss of H_2_ and PPh_3_ (Scheme 4).^[22]^ Green‐brown crystals of **6** were analysed by single‐crystal XRD to reveal a distorted octahedral Ru centre with *trans*‐hydrides and the two PPh_3_ ligands mutually *cis*‐, with the η^1^,η^1^‐P,P’‐chelating {Yb(THF)_2_(Tmp)_2_} metalloligand completing the Ru coordination sphere. The coordination of the Tmp P lone pairs to Ru enforces a near‐eclipsed configuration of the two C_4_P rings, which are bound in an η^5^,η^5^‐fashion to Yb in a bent metallocene motif (Yb−P: 2.930(2) Å), with two mutually *cis*‐THF molecules completing the Yb coordination sphere. The ^31^P NMR spectrum of **6** exhibited doublets for both the PPh_3_ and Tmp P atoms, with the latter signal of interest at 103 ppm, confirming that the 220 Hz splitting is due to a *trans*‐^2^ *J*_PP_ coupling, with no *J*_YbP_ coupling constants disclosed.

**Figure 6:**
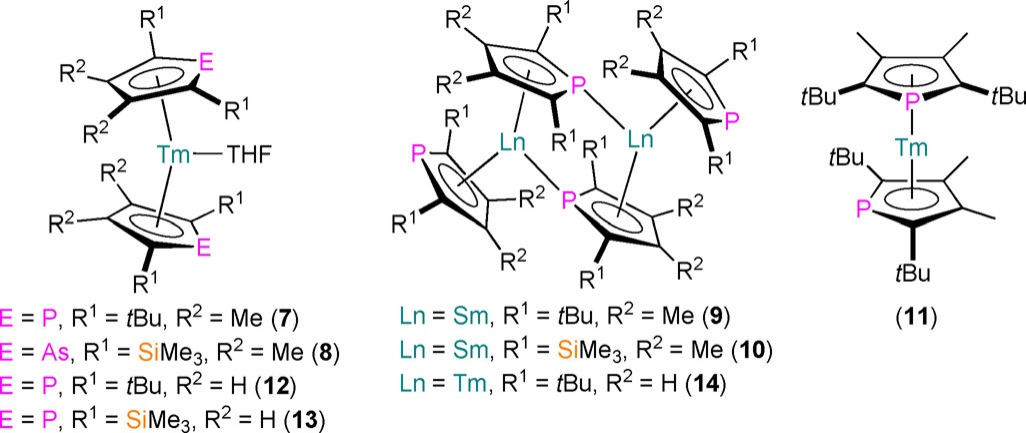
Complexes **7**–**14**.[^23^, ^25^, ^26^, ^27^]

In 2002, Nief and co‐workers extended Ln^II^ phospholyl and arsolyl chemistry to Tm, through the synthesis of [Tm(L)_2_(THF)] (L=η^5^‐Dtp, **7**; Dsp; η^5^‐Dsas, **8**; Figure 6) by salt metathesis protocols from TmI_2_(THF)_3_ and parent potassium salts in diethyl ether.^[23]^ The +2 oxidation state of Tm is rarely observed in molecular complexes,^[5]^ and at the time **7** and **8** were the only structurally authenticated organometallic Tm^II^ complexes in the literature apart from [Tm{C_5_H_3_(SiMe_3_)_2_‐1,3}_2_(THF)].^[24]^ The increased thermal stability of **7** and **8** over the previously reported Cp^R^ analogue highlights that the poorer π‐donor capabilities of phospholyl and arsolyl ligands are effective for stabilising low oxidation state metals, whilst the incorporation of bulkier substituents in the α‐positions in Dtp, Dsp and Dsas compared with Tmp provides additional kinetic stabilisation; however, **7** and **8** slowly decompose at room temperature under argon.^[23]^ The solid‐state structures of **7** and **8** were obtained, with both complexes adopting similar pseudo‐bent metallocene geometries in the solid state with two η^5^‐Dtp/Dsas ligands and a single THF molecule coordinated to the thulium centre; as expected the Tm−P distances of **7** (2.943(1) and 2.967(1) Å) are overall shorter than the Tm−As distances of **8** (2.968(1) and 2.9759(8) Å). The Tm^II^ phospholyl complexes in this paper were also characterised by ^31^P NMR spectroscopy (*δ*_P_: −338.3 for L=Dtp, **7**, and −265.7 ppm for L=Dsp).

In 2003, Nief and co‐workers reported the synthesis of donor solvent‐free [{Sm(η^5^‐Dtp)(μ:η^5^,η^1^‐Dtp)}_2_] (**9**), [{Sm(η^5^‐Dsp)(μ:η^5^,η^1^‐Dsp)}_2_] (**10**) and [Tm(η^5^‐Dtp)_2_] (**11**; Figure 6), and solvated [Tm(Dsp)_2_(OEt_2_)], by performing salt metathesis reactions in diethyl ether using the parent LnI_2_ and two equivalents of K(Dtp) or K(Dsp).^[25]^ The coordinated diethyl ether was easily removed under vacuum in all cases apart from [Tm(Dsp)_2_(OEt_2_)], as this solvent does not bind as strongly as THF. A combination of ligand effects and the size of the Ln^II^ centre influences both the tendency of the complexes to desolvate and whether or not oligomerisation occurs. In the solid state, **9** and **10** exhibit dinuclear structures with two η^5^‐ and two μ:η^5^,η^1^‐bound ligands owing to their relatively large Sm^II^ centres, with the longest Sm−P distances arising from the η^1^‐bound P atoms (Sm−P: 3.045(1), 3.148(1) and 3.197(1) Å for **9**; and 3.023(1), 3.113(1) and 3.168(1) Å for **10**). In contrast with the bent metallocene THF adduct **7**,^[23]^ complex **11** adopts a near‐linear geometry (Dtp_centroid_⋅⋅⋅Tm⋅⋅⋅Dtp_centroid_: 170°), exhibiting almost eclipsed C_4_P rings but with staggered P atoms (Tm−P: 2.867(2) and 2.875(2) Å).^[25]^ ^1^H NMR spectroscopy indicated that **9** and **10** are monomeric in C_6_D_6_ solution, and ^31^P NMR spectra were obtained in THF solutions, which show broad resonances that are likely of solvated monomers (*δ*_P_: −519 ppm for **9⋅THF**, −383 ppm for **10⋅THF**). Similarly, diethyl ether reaction mixtures for Tm^II^ analogues exhibited signals in their ^31^P NMR spectra at −310 ppm for **11⋅OEt_2_** and −273 ppm for [Tm(Dsp)_2_(OEt_2_)]; a signal at −257 ppm was reported for a C_6_D_6_ solution of donor solvent‐free **10**.

In follow‐up papers in 2005 and 2007, Nief and co‐workers reported crystallographic characterisation of the solvated Tm^II^ mononuclear complexes [Tm(Htp)_2_(THF)] (**12**) and [Tm(Hsp)_2_(THF)] (**13**),^[26]^ and the solvent‐free dinuclear Tm^II^ complex [{(η^5^‐Htp)Tm(μ:η^5^,η^1^‐Htp)}_2_] (**14**)^[27]^ (Figure 6), as part of investigations to compare the donor properties of Cp^R^ and phospholyl ligands in Tm^II^ chemistry. Complexes **12** and **13** were synthesised by analogous salt metathesis methods^[26]^ to those used for **7**–**11**,^[25]^ whilst **14** was prepared by reduction of the Tm^III^ precursor [{Tm(η^5^‐Htp)_2_(μ‐I)}_2_] with KC_8_ in hexanes.^[27]^ The monomeric complexes exhibited broad ^31^P NMR spectra (*δ*_P_: −290 for **12**, −235 ppm for **13**)^[26]^ and no signal was seen for **14** in C_6_D_6_ unless THF was added, whereby a ^31^P NMR spectrum identical to that of solvated **12** was observed.^[27]^ The pseudo‐bent metallocene structures of mononuclear **12** and **13** in the solid state are similar to those of **7** and **8**,^[23]^ with the lack of Me groups at the β‐positions leading to larger C_4_P_centroid_⋅⋅⋅Tm⋅⋅⋅C_4_P_centroid_ angles and shorter mean Tm−P distances (2.941(2) Å for **12** and 2.921(1) Å for **13**).^[26]^ The solid‐state structure of dinuclear **14** is analogous to **9** and **10**,^[25]^ with shorter Ln−P distances for **14** (2.954(2), 3.028(2) and 3.002(2) Å)^[27]^ owing to the smaller size of Tm^II^ versus Sm^II^.^[1a]^

**Figure 7:**
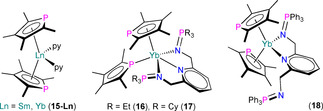
Complexes **15**–**18**.[^28^, ^29^]

Simultaneously to the disclosure of the solid structure of **14**, Nief and co‐workers stated that treatment of **11** with pyridine gave NMR spectra consistent with the formation of an adduct [Tm(Dtp)_2_(NC_5_H_5_)],^[27]^ but this product was not structurally authenticated. In 2012, Labouille et al. showed that [Sm(Tmp)_2_] reacted with pyridine to give the adduct [Sm(η^5^‐Tmp)_2_(py)_2_] (**15‐Sm**);^[28]^ the corresponding Yb^II^ complex **15‐Yb** was reported in 2015 by Nocton, Auffrant and Cheisson, together with the structures of several similar substituted Yb^II^ bis‐phospholyl complexes coordinated by substituted pyridines, [Yb(Tmp)_2_(L^R^)] (L^R^=C_5_H_3_N(CH_2_NPR_3_)_2_‐2,6; R=Et, **16**; Cy, **17**; Ph, **18**)^[29]^ (Figure 7). As both [Tm(Cp^ttt^)_2_]^[27]^ and [Sm(Cp*)_2_]^[28]^ reductively couple pyridine, the lack of SET chemistry of **11** and [Sm(Tmp)_2_] towards this substrate showcases how the weaker π‐donor properties of phospholyl ligands versus Cp^R^ makes metal centres less reducing. However, the next section shows that the SET chemistry of Ln^II^ phospholyl complexes is still rich and can exhibit considerable reduction potentials.

The solid‐state structures of **15‐Ln** for both Sm^[28]^ and Yb^[29]^ reveal bent metallocene‐type motifs, with two eclipsed η^5^‐Tmp ligands with staggered C_4_P rings (Sm−P: 3.043(1) and 3.046(1) Å; Yb−P: 2.903(8) and 2.941(2) Å) and two equatorially coordinated pyridine molecules. The structures of **16**–**18** differ according to the identity of L^R^, with **16** and **17** containing κ^3^‐coordinated L^R^ ligands to enforce one η^1^‐bound Tmp (Yb−P: 2.9723(8) Å for **16** and 3.023(1) Å for **17**) and one η^5^‐Tmp (Yb−P: 3.027(1) Å for **16** and 2.925(1) Å for **17**).^[29]^ Only one of the iminophosphoranyl arms are bound in the κ^2^‐L^Ph^ ligand of **18**, thus both Tmp ligands are able to bind in an η^5^‐fashion (Yb−P: 2.9480(8) and 2.947(1) Å). One broad signal was observed in the ^31^P NMR spectrum of paramagnetic **15‐Sm** (*δ*_P_: −624 ppm, *W*_1/2_=550 Hz),^[28]^ whereas coupling to ^171^Yb is observed in diamagnetic **15‐Yb** (*δ*_P_: 79.5 ppm, ^1^ *J*_YbP_=105.5 Hz).^[29]^ Similarly, only one signal was observed in the ^31^P NMR spectra of **16** (*δ*_P_: 77.9 ppm, ^1^ *J*_YbP_=434.7 Hz) and **17** (*δ*_P_: 79.8 ppm, ^1^ *J*_YbP_=491.4 Hz) at 298 K, which the authors assigned to the Tmp ligands both being η^1^‐bound at this temperature owing to the large coupling constants. Variable‐temperature studies showed that these signals decoalesce at −90 °C, whereas the κ^3^‐binding mode of L^R^ persisted in solution for **16** and **17** at all temperatures investigated. In contrast, the ^31^P NMR spectrum of **18** exhibits a signal at 79.0 ppm with a coupling constant more in line with η^1^‐Tmp (^1^ *J*_YbP_=172.3 Hz), and the asymmetrically‐bound κ^2^‐L^Ph^ showed two phosphorus environments.

### 5.2 Ln^III^ complexes

#### 5.2.1 Ln^III^ complexes derived from Ln^II^ precursors

**Scheme 5:**
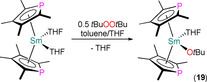
Synthesis of **19** by the SET reaction of [Sm(Tmp)_2_(THF)_2_] with 0.5 equivalents of *t*BuOO*t*Bu.^[30]^

Redox reactions of Ln^II^ complexes over the last two decades have furnished a number of structurally authenticated Ln^III^ monophospholyl products, which we cover here. In 2001, Barbier‐Baudry et al. reported the reaction of [Sm(Tmp)_2_(THF)_2_] with half an equivalent of *tert*‐butylperoxide to give [Sm(η^5^‐Tmp)_2_(O*t*Bu)(THF)] (**19**) by an SET reaction in a toluene/THF mixture (Scheme 5).^[30]^ Orange crystals of **19** were studied by XRD to reveal a pseudo‐bent metallocene geometry at the Sm^III^ centre with staggered η^5^‐Tmp rings (Sm−P: 2.951(2) and 3.026(2) Å) and both a *tert*‐butoxide and a THF molecule coordinating at the equatorial positions. Complex **19** was shown to be an effective initiator for the ring‐opening polymerisation of *ϵ*‐caprolactone in the same publication.

**Figure 8:**
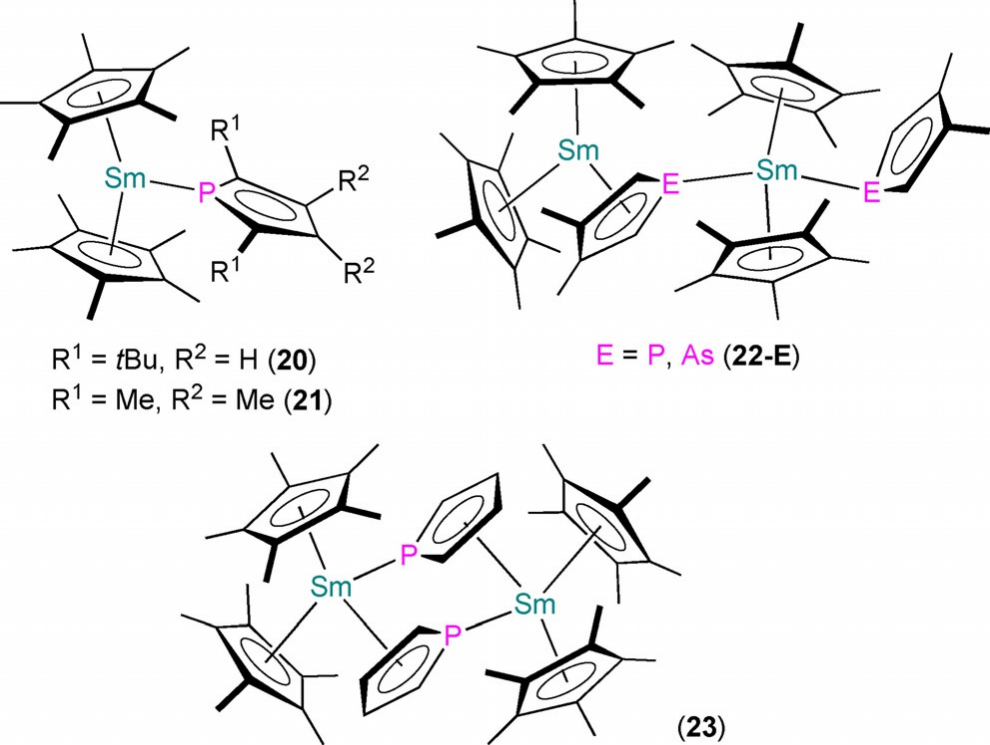
Complexes **20**–**23**.^[31]^

Also in 2001, Nief and Ricard reported the syntheses of a series of bis‐Cp* Sm^III^ complexes bound by phospholyl or arsolyl ligands: [Sm(Cp*)_2_(η^5^‐Htp)] (**20**), [Sm(Cp*)_2_(η^1^‐Tmp)] (**21**), [Sm(Cp*)_2_(μ:η^5^,η^1^‐Mhp)Sm(Cp*)_2_(η^1^‐Mhp)] (**22‐P**), [Sm(Cp*)_2_(μ:η^5^,η^1^‐Mhas)Sm(Cp*)_2_(η^1^‐Mhas)] (**22‐As**) and [{Sm(Cp*)_2_(μ:η^5^,η^1^‐C_4_H_4_P)}_2_] (**23**; Figure 8).^[31]^ Complexes **20**–**22** were synthesised by the separate SET reactions of [Sm(Cp*)_2_] or [Sm(Cp*)_2_(OEt_2_)] with the parent biphosphole or biarsole, whereas **23** was most straightforwardly prepared from [Sm(Cp*)_2_(OEt_2_)] and [Tl(C_4_H_4_P)]. The solid‐state structures of **20**–**23** vary with the steric requirements of the various phospholyl and arsolyl ligands. Complexes **20** and **21** are monomeric, with an η^5^‐Htp in the former (Sm−P: 3.153(1) Å) and an unsymmetrically bound η^1^‐Tmp in the latter, with two independent molecules in the asymmetric unit showing different Sm−P distances (2.856(1) and 2.891(1) Å). Complexes **22‐E** and **23** are dinuclear with bridging phospholyl or arsolyl ligands; for the former examples, these asymmetric dimers each contain one μ:η^5^,η^1^‐Mhp (Sm−P: 3.0132(8) and 3.1032(8) Å) or μ:η^5^,η^1^‐Mhas (Sm−As: 3.0671(6) and 3.1610(6) Å), and one η^1^‐Mhp (Sm−P: 2.886(1) Å) or η^1^‐Mhas (Sm−As: 2.9776(8) Å) bound to the less congested Sm^III^ centre. Complex **23** is a symmetric dimer with two μ:η^5^,η^1^‐C_4_H_4_P bridges (Sm−P: 3.101(2) and 3.274(1) Å), with each Sm^III^ centre showing identical coordination spheres.

**Figure 9:**
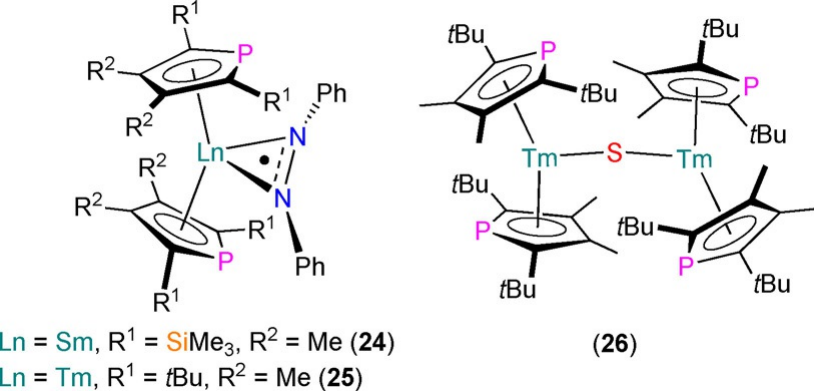
Complexes **24**–**26**.^[25]^

In 2003, Nief and co‐workers treated **9**–**11** separately with azobenzene to give mononuclear Ln^III^ complexes bound by azobenzenyl radicals following SET; single‐crystal XRD data were obtained for [Sm(η^5^‐Dsp)_2_(N_2_Ph_2_)] (**24**) and [Tm(η^5^‐Dtp)_2_(N_2_Ph_2_)] (**25**; Figure 9), but not for [Sm(Dtp)_2_(N_2_Ph_2_)].^[25]^ Complexes **24** and **25** exhibit similar open‐metallocene type structures with η^5^‐bound phospholyl ligands (Ln−P: 2.9484(6) Å for **24** and 2.869(1) Å for **25**) and equatorially bound η^2^‐N_2_Ph_2_ radicals, with the expected differences in metrical parameters arising from varying Ln^III^ ionic radii and ring substitution. The ^31^P NMR spectra for **24** (*δ*_P_: 148 ppm) and [Sm(Dtp)_2_(N_2_Ph_2_)] (*δ*_P_: 46 ppm) showed vastly different chemical shifts owing to paramagnetic effects. In the same paper, **9**–**11** were treated separately with half an equivalent of triphenylphosphine sulfide, but an SET reaction was only observed for the Tm analogue to afford the dinuclear Tm^III^ complex [{Tm(η^5^‐Dtp)_2_}_2_(μ‐S)] (**26**; Figure 9), with concomitant loss of triphenylphosphine. The solid‐state structure of **26** revealed mean Tm−P distances of 2.875(7) Å for the η^5^‐Dtp ligands, with a bent Tm‐S‐Tm motif (165.3(2)°).

**Figure 10:**
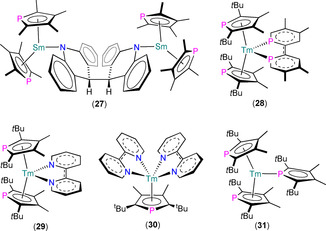
Complexes **27**–**31**.[^28^, ^32^, ^33^]

Several other Ln^III^ phospholyl complexes have been shown to form via SET reactions of Ln^II^ precursors (Figure 10). In 2012, Labouille et al. showed that the dinuclear Sm^III^ complex [{Sm(η^5^‐Tmp)_2_}_2_(μ‐NC_13_H_9_‐C_13_H_9_N)] (**27**) formed from the reductive coupling of acridine by [Sm(Tmp)_2_].^[28]^ Complex **27** exhibits asymmetrical Sm^III^ coordination spheres (range Sm−P: 2.889(5)–2.937(5) Å) with staggered bent {Sm(η^5^‐Tmp)_2_} fragments bound by the bridging dianionic (C_13_H_9_N)_2_ ligands through the N‐atoms; ^31^P NMR spectroscopy revealed two resonances with *W*_1/2_ of 80 Hz (*δ*_P_: 43.24 and 46.75 ppm), indicating that this asymmetry is maintained in the solution phase. In 2014, Clavaguéra, Nocton and co‐workers reacted **11** with tetramethyl‐2,2’‐bisphosphinine (tmbp) and 2,2’‐bipyridine (bipy) to yield the Tm^III^ radical complexes [Tm(η^5^‐Dtp)_2_(tmbp)] (**28**) and [Tm(η^5^‐Dtp)_2_(bipy)] (**29**), respectively.^[32]^ The reaction of **28** with bipy gave **29** with the elimination of tmbp, and the reaction of **29** with a second equivalent of bipy gave a complex with two bipy**^.^** radicals, [Tm(η^5^‐Dtp)(bipy)_2_] (**30**), by the reductive coupling of Dtp to give half an equivalent of (Dtp)_2_. Although the paramagnetism of **28**–**30** prevented interpretable ^31^P NMR data from being obtained, all three complexes were characterised by single‐crystal XRD. The solid‐state structures of **28** and **29** are similar, with two η^5^‐Dtp (Tm−P: 2.825(2) and 2.862(2) Å for **28** and 2.843(2) and 2.844(2) Å for **29**) and a bidentate equatorially bound radical ligand. The Tm^III^ centre in **30** only contains one η^5^‐Dtp (Tm−P: 2.841(2) Å) and two bidentate bipy**^.^** radicals. Complexes **28**–**30** were subjected to detailed magnetic and computational studies to establish their electronic structures. Finally, in 2016, Jaroschik, Nocton and co‐workers reported the separate SET reactions of [Tm(Cp^ttt^)_2_] and **11** with half an equivalent of [Pb(Dtp)_2_]; “[Tm(Cp^ttt^)_2_][Dtp]” was characterised in the former reaction but no solid‐state structure could be obtained, whereas [Tm(η^5^‐Dtp)_2_(η^1^‐Dtp)] (**31**) from the latter reaction was structurally authenticated.^[33]^ The Tm^III^ centre of **31** is bound by two η^5^‐ (Tm−P: 2.8600(7) and 2.8727(7) Å) and one η^1^‐ (Tm−P: 2.8135(8) Å) Dtp. Variable‐temperature ^1^H NMR spectra of **31** indicated that the two η^5^‐Dtp are bound in solution at room temperature, with rapid exchange of the η^1^‐Dtp; cooling the solution to below −50 °C revealed a third resonance, which was assigned as the bound η^1^‐Dtp by shifting this dynamic equilibrium towards the observed solid‐state structure.

#### 5.2.2 Ln^III^ complexes derived from Ln^III^ starting materials

##### 5.2.2.1 Complexes containing only halide co‐ligands or no co‐ligand

**Figure 11:**
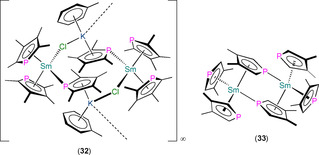
Complexes **32** and **33**.^[34]^

The first report of Ln^III^ halides being used to synthesise phospholyl complexes was by Nief and Mathey in 1989, where the authors presented the salt metathesis reactions of LnCl_3_ (Ln=Y, Lu) with two equivalents of Li(Tmp) in ethereal solvents to give [Ln(Tmp)_2_(μ‐Cl)_2_Li(Sol)_2_] (Ln=Y, Sol=DME; Ln=Lu, Sol=OEt_2_).^[10]^ These diamagnetic Ln^III^ complexes were characterised by multinuclear NMR spectroscopy, with each showing one signal in their ^31^P NMR spectra (*δ*_P_: 84.0 ppm, ^1^ *J*_YP_=6.4 Hz for Ln=Y; *δ*_P_: 78.6 ppm for Ln=Lu), with ^89^Y NMR data used to corroborate the coupling in the former complex and assign η^5^‐bound Tmp ligands, but no solid‐state structures were obtained. In 1995, Nief and co‐workers reported the synthesis of the Sm^III^ phospholyl complexes [{Sm(η^5^‐Tmp)(μ:η^5^,η^1^‐Tmp)_2_(μ‐Cl)K(C_7_H_8_)}_2_]_∞_ (**32**) and [{Sm(η^5^‐Mhp)_2_(μ:η^5^,η^1^‐Mhp)}_2_] (**33**; Figure 11) by the reactions of SmCl_3_ with three equivalents of the respective group 1 ligand transfer agents K(Tmp) and K(Mhp) in toluene at reflux.^[34]^ The solid‐state structure of salt‐occluded **32** revealed two crystallographically distinct Sm^III^ centres, each with one terminal η^5^‐Tmp (Sm−P: 2.953(3) Å), a μ:η^5^,η^1^‐Tmp that is η^5^‐bound to Sm (Sm−P: 2.924(4) Å) and a μ:η^5^,η^1^‐Tmp that is η^1^‐bound to Sm (Sm−P: 2.931(4) Å); a chloride completes the Sm coordination spheres that also bridge to K, which are in turn η^6^‐bound by toluene and η^5^‐bound by bridging phospholyls to give a net‐like structure. The Sm^III^ centres in salt‐free dinuclear **33** are each η^5^‐bound to three Mhp ligands and η^1^‐bound to a fourth, as a consequence of two asymmetrically bound μ:η^5^,η^1^‐Mhp ligands (Sm−P: 2.9270(5), 2.9862(5) and 2.9978(5) Å). The ^31^P NMR spectrum of **32** in C_7_D_8_ exhibited six broad signals (*δ*_P_: 34.1, 41.4, 44.5, 47.7, 50.1 and 52.3 ppm; *W*_1/2_ range: 12–109 Hz), indicating that a number of different species are present in solution of varying aggregation, whereas the ^31^P NMR spectrum of **33** in C_4_D_8_O showed one signal (*δ*_P_: 9.3 ppm, *W*_1/2_=120 Hz), indicating that monomeric [Sm(η^5^‐Mhp)_3_(C_4_D_8_O)] formed in solution.

**Figure 12:**
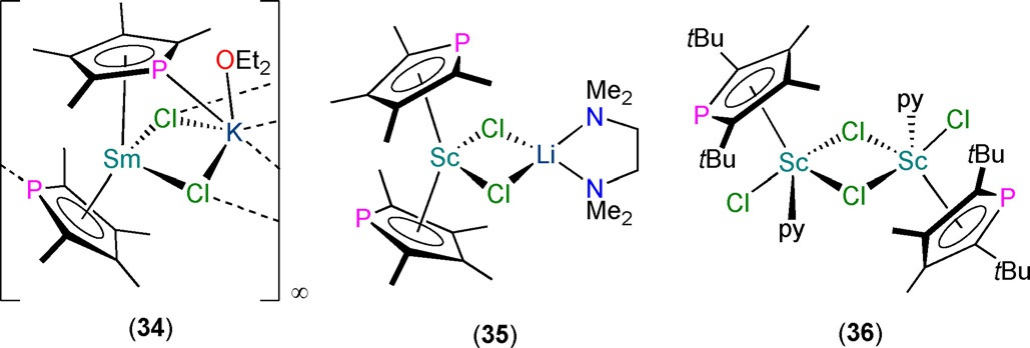
Complexes **34**–**36**.[^35^, ^36^, ^37^]

Several other heteroleptic Ln^III^ Tmp chloride complexes have been reported in the interim (Figure 12). In 1999, Nief and co‐workers prepared the ‘ate’ complexes [Ln(Tmp)_2_Cl_2_K] (Ln=Nd, Sm) by the separate reactions of parent LnCl_3_(THF)_*n*_ with two equivalents of K(Tmp) in THF; recrystallisation of the Sm analogue from diethyl ether allowed the solid‐state structure of [Sm(η^5^‐Tmp)(μ:η^5^,η^1^‐Tmp)(μ_3_‐Cl)_2K(_Et_2_O)]_∞_ (**34**) to be determined.^[35]^ Complexes [Ln(Tmp)_2_Cl_2_K] were characterised by ^31^P NMR spectroscopy (Ln=Nd, *δ*_P_: 459 ppm; Ln=Sm, *δ*_P_: 49.7 ppm). In the solid state, the Sm^III^ centres of polymeric **34** exhibit open metallocene‐type motifs with two η^5^‐Tmp ligands (Sm−P: 2.905(2) and 2.926(1) Å), one of which is also η^1^‐bound to K, with two chlorides bridging to multiple K atoms, which are capped with a single diethyl ether molecule. In 2006, Tilley and co‐workers reported the synthesis of [Sc(η^5^‐Tmp)_2_(μ‐Cl)_2_Li(TMEDA)] (**35**, TMEDA = *N*,*N*,*N*’,*N*’‐tetramethylethylenediamine) by the reaction of ScCl_3_(THF)_3_ with two equivalents of Li(Tmp)(TMEDA) in toluene.^[36]^ The ^31^P NMR spectrum of **35** exhibits one signal at 99.8 ppm, and the local structure about Sc^III^ in the solid state is comparable to the Sm^III^ centre in **34**,^[35]^ although the alteration of coordinating solvent, alkali metal and Ln^III^ ion enforces a monomeric structure at **35** (Sc−P: 2.694(2) and 2.718(2) Å).^[36]^ The separate reactions of **35** with LiCp* or Sc(Cp*)(Cl)_2_ with Li(Tmp)(TMEDA) both gave reaction mixtures with signals in their ^31^P NMR spectra at 100.2 ppm, which the authors ascribed to the mixed Cp*/Tmp complex [Sc(Cp*)(Tmp)(μ‐Cl)_2_Li(TMEDA)], although this product was not structurally authenticated. Finally, in 2007, Nief, Hou and co‐workers reported the synthesis of dinuclear [{Sc(η^5^‐Dtp)(μ‐Cl)(Cl)(py)}_2_] (**36**) from the equimolar reaction of ScCl_3_ and K(Dtp) in a mixture of toluene and pyridine (5:1).^[37]^ Complex **36** exhibits a single peak in its ^31^P NMR spectrum (123.0 ppm), and single‐crystal XRD revealed each Sc^III^ centre is bound by a molecule of pyridine, a terminal chloride and two bridging chlorides, and is capped by one η^5^‐Dtp (Sc−P: 2.6960(5) Å).

**Figure 13:**
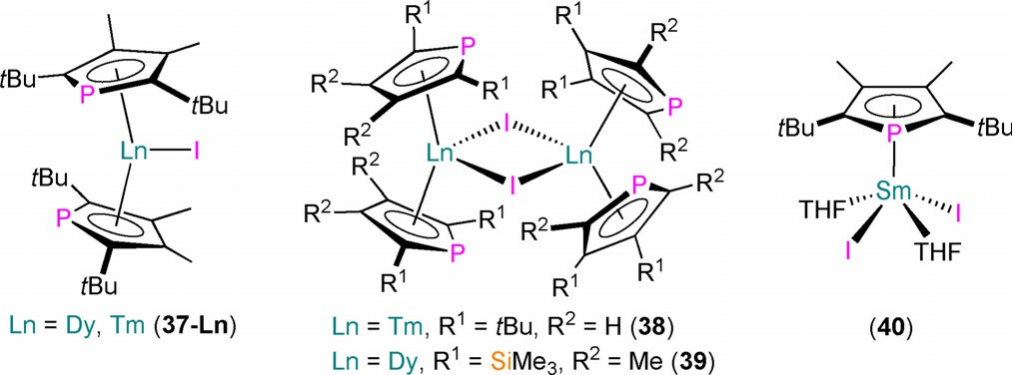
Complexes **37**–**40**.[^27^, ^37^, ^38^]

A handful of Ln^III^ phospholyl iodide complexes have also been structurally authenticated (Figure 13). In 2007, Nief and co‐workers reported the synthesis of mononuclear [Tm(η^5^‐Dtp)_2_(I)] (**37‐Tm**) and dinuclear [{Tm(η^5^‐Htp)_2_(μ‐I)}_2_] (**38**) by the reaction of TmI_3_ with two equivalents of K(Dtp) or K(Htp) in toluene at reflux.^[27]^ In 2009, a Dy^III^ homologue **37‐Dy** and a related dinuclear complex [{Dy(η^5^‐Dsp)_2_(μ‐I)}_2_] (**39**) were reported to form by analogous methods.^[38]^ Complexes **37**–**39** all contain two η^5^‐bound phospholyl ligands at each metal centre; for mononuclear **37‐Ln** (Ln−P: 2.9235(2) Å for Dy,^[38]^ 2.8119(12) and 2.8167(12) Å for Tm^[37]^) the metals are also bound by a single iodide ligand, whereas dinuclear **38** (Tm−P: 2.906(2) and 2.9504(14) Å) and **39** (range Dy−P: 2.8500(7)–2.8690(7) Å) each exhibit two bridging iodides, which saturate their metal coordination spheres. Finally, in 2007, Nief, Hou and co‐workers disclosed the synthesis of the mono‐ring Sm^III^ complex [Sm(η^5^‐Dtp)(I)_2_(THF)_2_] (**40**) by the reaction of SmI_3_(THF)_3.5_ with equimolar K(Dtp).^[37]^ Complex **40** exhibits one signal in its ^31^P NMR spectrum at 77.5 ppm and has a typical half‐sandwich structure in the solid state, with mutually *trans*‐THF and iodide ligands and a single η^5^‐Dtp (Sm−P: 2.9112(14) Å) coordinated to Sm.

**Scheme 6:**
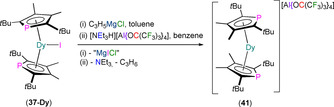
Synthesis of **41** by the sequential reaction of **37‐Dy** with Mg(C_3_H_5_)Cl and [NEt_3_H][Al{OC(CF_3_)_3_}_4_].^[39]^

The homoleptic mononuclear Dy^III^ complex [Dy(η^5^‐Dtp)_2_][Al{OC(CF_3_)_3_}_4_] (**41**) was synthesised in 2019 by Chilton, Mills and co‐workers by the sequential salt metathesis and protonolysis reaction of **37‐Dy** with allyl magnesium chloride and [NEt_3_H][Al{OC(CF_3_)_3_}_4_], with the respective elimination of magnesium dihalides, triethylamine and propylene providing thermodynamic driving forces (Scheme 6).^[39]^ The installation of a sufficiently weakly coordinating anion provided an isolated bent [Dy(η^5^‐Dtp)_2_]^+^ cation in the solid state, with Dy−P distances of 2.7880(8) and 2.7981(8) Å. The axial ligand field and rigidity of the aromatic ligands of **41** are both conducive to enhance the SMM properties for Dy^III^, and the effective barrier to magnetic reversal (1760 K) and maximum hysteresis temperature (48 K) for **41** are both competitive with leading Cp^R^ Ln SMMs.^[7]^

##### 5.2.2.2 Complexes containing borohydride co‐ligands

**Figure 14:**
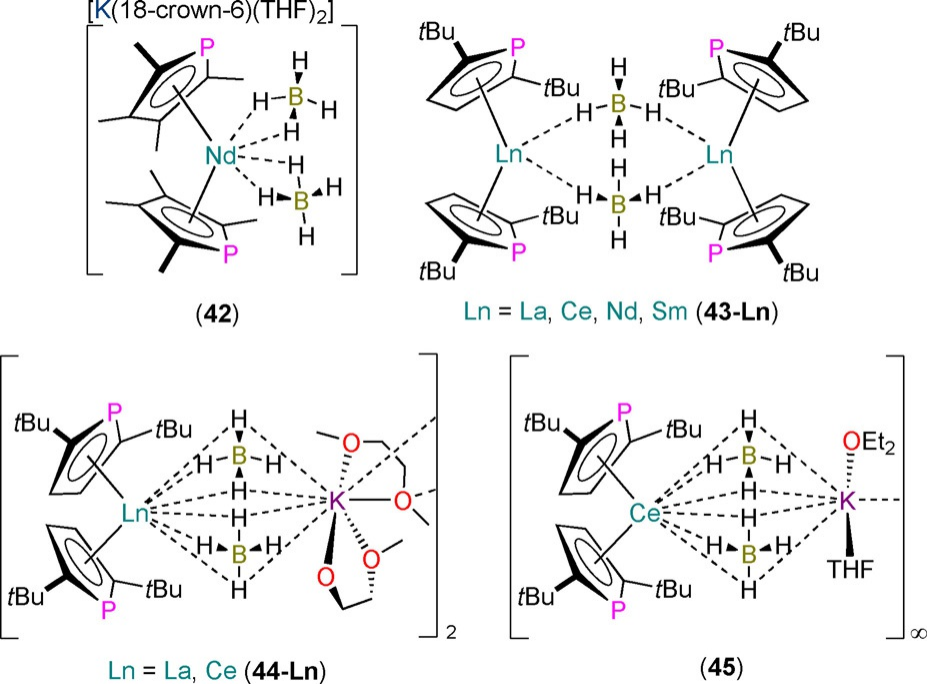
Complexes **42**–**45**.[^40^, ^41^, ^42^]

There have only been several heteroleptic Ln^III^ phospholyl borohydride complexes that have been structurally characterised to date (Figure 14). In 2000, Cendrowski‐Guillaume et al. reported the synthesis of the ‘ate’ complex “[K(THF)_*n*_][Nd(Tmp)_2_(BH_4_)_2_]” from the reaction of Nd(BH_4_)_3_(THF)_3_ with two equivalents of K(Tmp) in THF; although no solid‐state structure was obtained, the ^31^P NMR spectrum of this product contained one signal at 413 ppm.^[40]^ Two years later, the same authors reported that the addition of 18‐crown‐6 to this reaction mixture allowed the solid‐state structure of monomeric [K(18‐crown‐6)(THF)_2_][Nd(η^5^‐Tmp)_2_(BH_4_)_2_] (**42**) to be determined; the separated anion contains a pseudo‐tetrahedral Nd^III^ centre with two borohydride and two η^5^‐Tmp ligands (Nd−P: 2.982(3) and 2.995(3) Å).^[41]^ In 2020, Mills and co‐workers reported a series of salt metathesis reactions of THF solvates of light Ln^III^ borohydrides with K(Htp) in a range of stoichiometries and solvents to afford the polynuclear heteroleptic Ln^III^ phospholyl borohydride complexes [{Ln(η^5^‐Htp)_2_(μ‐BH_4_)}_2_] (**43‐Ln**; Ln=La, Ce, Nd, Sm) and [Ln(η^5^‐Htp)_2_(μ‐BH_4_)_2_K(Sol)_2_]_*n*_ (**44‐Ln**, Ln=La, Ce, Sol=2 DME, *n=*2; **45**, Ln=Ce, Sol=Et_2_O and THF, *n*=∞).^[42]^ The varying but similar local pseudo‐tetrahedral coordination spheres of {Ln(η^5^‐Htp)_2_(BH_4_)_2_} fragments in **43**–**45** were established by single‐crystal XRD (Ln−P: 3.089(5) and 3.138(3) Å, **43‐La**; 3.058(5) and 3.099(4) Å, **43‐Ce**; 3.019(11) and 3.077(6) Å, **43‐Nd**; 3.016(6) and 3.054(4) Å, **43‐Sm**; 3.1790(7) and 3.1869(7) Å, **44‐La**; 3.1456(13) and 3.1534(13) Å, **44‐Ce**; 3.1488(13) and 3.1682(12) Å, **45**). The paramagnetism of the majority of **43**–**45** precluded the collection of reliable ^31^P NMR spectra in most cases, but signals were observed for diamagnetic **43‐La** (*δ*_P_: 105.65 ppm) and **44‐La** (*δ*_P_: 96.49 ppm).

##### 5.2.2.3 Complexes containing alkyl co‐ligands

**Figure 15:**
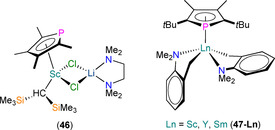
Complexes **46** and **47‐Ln** (Ln=Sc, Y, Sm).[^35^, ^36^, ^37^]

A handful of structurally authenticated heteroleptic Ln^III^ phospholyl alkyl complexes have been reported (Figure 15). In 1999, Nief et al. reported the syntheses of the bis‐phospholyl complexes [Ln(Tmp)_2_{CH(SiMe_3_)_2_}] (Ln=Nd, Sm), with the most efficient synthetic route being the sequential salt metathesis reactions of parent LnCl_3_(THF)_*n*_ with two equivalents of K(Tmp) followed by one equivalent of Li{CH(SiMe_3_)_2_} in THF.^[35]^ Although these products were not characterised by single‐crystal XRD, they each exhibited two signals in their ^31^P NMR spectra (*δ*_P_: 456 and 501 ppm for Ln=Nd; 43.4 and 46.7 ppm for Ln=Sm). Hydrogenolysis reactions of [Ln(Tmp)_2_{CH(SiMe_3_)_2_}] gave products with formulations consistent with reduction for Sm to form “[Sm(Tmp)_2_]”, and with σ‐bond metathesis for Nd to give “[Nd(Tmp)_2_(H)]”. Two Sc^III^ ‘ate’ complexes [Sc(Tmp)_2_(Me)(X)Li(TMEDA)] (X=Cl, Me) were reported in 2006 by Tilley and co‐workers, together with the first structurally characterised Ln^III^ phospholyl alkyl complex, [Sc(η^5^‐Tmp){CH(SiMe_3_)_2_}(μ‐Cl)_2_Li(TMEDA)] (**46**).^[36]^ These complexes were prepared by salt metathesis reactions of **35** with MeLi or Li{CH(SiMe_3_)_2_. Complexes [Sc(Tmp)_2_(Me)(X)Li(TMEDA)] (X=Cl, Me) could not be isolated but were assigned to signals in the ^31^P NMR spectra of reaction mixtures (*δ*_P_: 88.1 ppm for X=Cl, 82.9 for X=Me), whilst **46** exhibits *δ*_P_: 119.2 ppm. Single‐crystal XRD revealed a piano‐stool geometry about Sc, with the two chlorides bridging to Li, and η^5^‐Tmp (Sc−P: 2.712(2) Å). A family of heteroleptic Ln^III^ phospholyl complexes containing substituted benzyl ligands, [Ln(η^5^‐Dtp)(κ^2^‐CH_2_C_6_H_4_NMe_2_‐*o*)_2_] (**47‐Ln**, Ln=Sc, Y, Sm), were reported by Nief, Hou and co‐workers in 2007 by sequential salt metathesis reactions of parent LnCl_3_ with K(Dtp) and two equivalents of [K(CH_2_C_6_H_4_NMe_2_‐*o*)].^[37]^ Complexes **47‐Ln** exhibited signals in their ^31^P NMR spectra at 99.0 (Sc), 88.9 (Y) and 62.9 (Sm) ppm, and similar solid‐state structures with the benzyl ligands additionally coordinating with pendant NMe_2_ groups in a mutually *trans*‐fashion, and η^5^‐coordinated Dtp (Ln−P: 2.769(1), 2.928(1) and 3.009(1) Å for Sc, Y and Sm, respectively). When activated with [CPh_3_][B(C_6_F_5_)_4_], **47‐Sc** was shown to be efficient in promoting the formation of syndiotactic polystyrene, with **47‐Y** less efficient and **47‐Sm** unreactive towards styrene under identical conditions.

**Figure 16:**
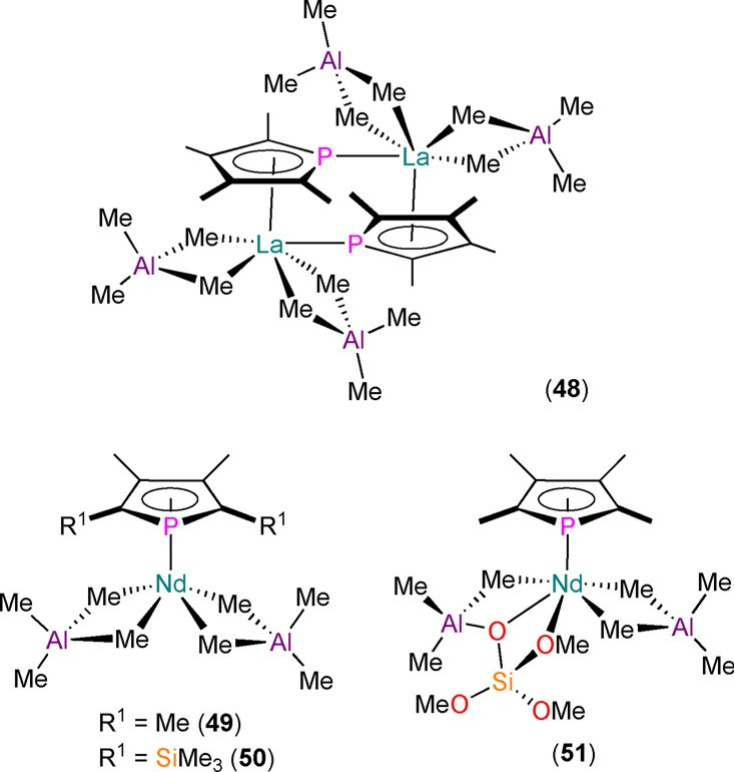
Complexes **48**–**51**.[^43^, ^44^]

A handful of heteroleptic Ln^III^ phospholyl aluminate complexes have been synthesised by Anwander and co‐workers (Figure 16).[^43^, ^44^] In 2007, the mono‐ring complexes [{La(μ:η^5^,η^1^‐Tmp)(AlMe_4_)_2_}_2_] (**48**), [Nd(η^5^‐Tmp)(AlMe_4_)_2_] (**49**), [La(Dsp)(AlMe_4_)_2_] and [Nd(η^5^‐Dsp)(AlMe_4_)_2_] (**50**) were reported to form via salt metathesis reactions of Ln(AlMe_4_)_3_ precursors with K(Tmp) or K(Dsp).^[43]^ All complexes exhibited one signal in their ^31^P NMR spectra (*δ*_P_: 128.4, **48**; 198.0, [La(Dsp)(AlMe_4_)_2_]; 444.0, **49**; 484.1 ppm, **50**). The contrasting solid‐state structures of dinuclear **48** and mononuclear **49** were attributed to differing Ln^III^ charge densities, with the former complex exhibiting both η^1^‐ (La−P: 3.1962(3) Å) and η^5^‐ (La−P: 3.0604(3) Å) binding modes of the bridging Tmp rings. Complexes **49** (Nd−P: 2.9252(10) Å) and **50** (Nd−P: 2.8972(3) Å) exhibited mononuclear half‐sandwich motifs with η^5^‐bound phospholyls; a crystal structure was not obtained for [La(Dsp)(AlMe_4_)_2_]. In follow‐up work in 2012, **48**–**50** and [La(Dsp)(AlMe_4_)_2_] were reacted with silica, and the resultant materials were investigated as catalysts in isoprene polymerisation.^[44]^ As part of this work, [Nd(η^5^‐Tmp)(AlMe_4_){OSi(O*t*Bu)_3_(AlMe_3_)}] (**51**) was synthesised by the protonolysis reaction of **49** with HOSi(O*t*Bu)_3_ to provide a molecular complex that models the surface species formed on silica. Complex **51** exhibits a signal at 544 ppm in its ^31^P NMR spectrum, and its solid‐state structure is comparable to that of **49**, with a Nd−P distance of 2.9652(6) Å.

##### 5.2.2.4 Complexes containing a cyclooctatetraenyl co‐ligand

**Figure 17:**
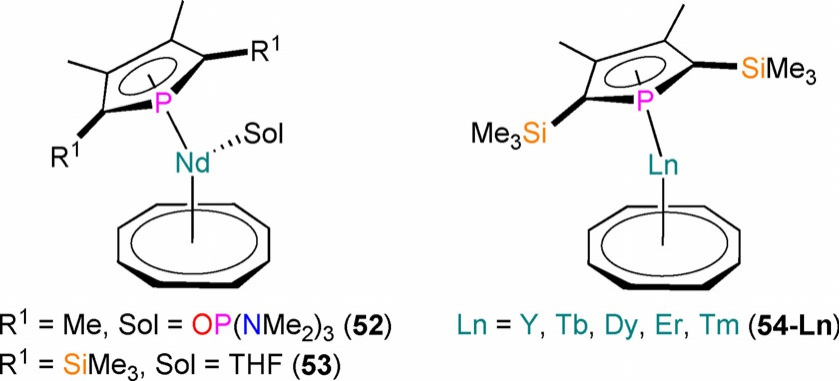
Complexes **52**–**54**.[^46^, ^47^, ^48^]

Mixed sandwich Ln^III^ phospholyl complexes are rare, and apart from the Cp^R^ examples in prior sections there are several other structurally authenticated complexes containing cyclooctatetraenyl ligands (C_8_H_8_, COT) (Figure 17). In 2002, Cendrowski‐Guillaume et al. reported that the reaction of [Nd(COT)(BH_4_)(THF)_2_] with K(Tmp) gave a powder formulated as “[Nd(COT)(Tmp)(THF)]”; exposure of this powder to vacuum removed the THF and the residue was formulated as a dinuclear complex, “[Nd(COT)(Tmp)]_2_”.^[45]^ The same solvent‐free complex was later obtained by the reaction of [Nd(COT)(THF)_4_][BPh_4_] with K(Tmp), and subsequent reaction with hexamethylphosphoramide (HMPA) gave green crystals of [Nd(COT)(η^5^‐Tmp)(HMPA)] (**52**).^[46]^ Complex **52** exhibits an open metallocene‐type geometry with a Nd−P distance of 2.968(8) Å; this structure is analogous to a complex reported the previous year by Visseaux and co‐workers, [Nd(COT)(η^5^‐Dsp)(THF)] (**53**), although this exhibits a longer Nd−P distance (3.1095(4) Å).^[47]^ Complex **53** was synthesised from the reaction of [Nd(COT)(μ‐Cl)(THF)_2_]_2_ with two equivalents of K(Tmp); the Sm^III^ complexes [Sm(COT)(Tmp)(THF)] and [Sm(COT)(Dsp)] were accessed by similar methods and although these were not structurally authenticated, the Tmp analogue was found to exhibit a signal in its ^31^P NMR spectrum at 134.1 ppm. In 2018, Chen et al. adapted these methods to synthesise [Ln(COT)(η^5^‐Dsp)] (**54‐Ln**, Ln=Y, Tb, Dy, Er, Tm) from [Ln(COT)(I)(THF)_2_] precursors.^[48]^ Complexes **54‐Ln** do not contain a coordinated THF molecule as late Ln have smaller ionic radii;^[1a]^ these mixed metallocene complexes exhibit Ln−P bonds lengths of 2.8261(6) (Y), 2.8745(12) (Tb), 2.8577(13) (Dy), 2.7929(11) (Er) and 2.7823(12) (Tm) Å.^[48]^ The Y(III) complex **54‐Y** exhibits a doublet in its ^31^P NMR spectrum at 157.96 ppm (^1^ *J*_YP_=12.1 Hz), whilst the Er^III^ analogue **54‐Er** has a favourable geometry for enhanced SMM properties, and was found to exhibit a competitive barrier to magnetic reversal of 367 K.

## 6 Actinide Monophospholyl and ‐arsolyl Complexes

### 6.1 An^III^ complexes

**Scheme 7:**
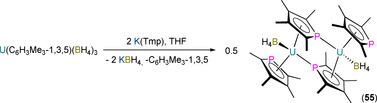
Synthesis of **55** from [U(C_6_H_3_Me_3_‐1,3,5)(BH_4_)_3_] and 2 equivalents of K(Tmp).[^49^, ^50^, ^51^]

There are only a handful of examples of An monophospholyl complexes, and only one monoarsolyl complex, that have been structurally authenticated to date, and to the best of our knowledge all reported An examples are of uranium. Baudry, Nief and co‐workers reported the first An monophospholyl complexes in 1990, where it was disclosed that the salt metathesis reaction of [U(C_6_H_3_Me_3_‐1,3,5)(BH_4_)_3_] with two equivalents of K(Tmp) in THF yielded the ‘ate’ complex “[U(Tmp)_2_(BH_4_)_2_]K”; subsequent removal of THF and addition of toluene to the reaction mixture gave the dinuclear U^III^ complex [U(η^5^‐Tmp)(μ;η^5^.η^1^‐Tmp)(BH_4_)]_2_ (**55**; Scheme 7).^[49]^ Complex **55** exhibited two broad signals in its ^31^P NMR spectrum at *δ*_P_: 727 (*W*_1/2_=150 Hz) and 3471 (*W*_1/2_=1000 Hz) ppm, and reacted separately with THF and OPPh_3_ to give the monomeric Lewis base adducts [U(Tmp)_2_(BH_4_)(THF)] and [U(Tmp)_2_(BH_4_)(OPPh_3_)], respectively. No U^III^ complexes were structurally authenticated in this initial report.

In a follow‐up full paper in 1992, Gradoz et al. presented an alternative synthetic route to obtain **55** by Na/Hg amalgam reduction of the U^IV^ precursor [U(Tmp)_2_(BH_4_)_2_] in toluene (see Section 6.2).^[50]^ Two U^III^ ‘ate’ complexes, [Na(15‐crown‐5)][U(Tmp)(BH_4_)_3_] and [Na(15‐crown‐5)][U(Tmp)_2_(BH_4_)_2_], were also reported in this paper to form from analogous Na/Hg reductions of the respective U^IV^ precursors [U(Tmp)(BH_4_)_4_] and [U(Tmp)_2_(BH_4_)_2_] in the presence of 15‐crown‐5, but these products were characterised by elemental analysis only. The ^1^H NMR spectrum of **55** was investigated in additional detail, and variable‐temperature experiments were performed to determine that a *cis*‐/*trans*‐isomerisation equilibrium is in operation in solution owing to fluxional η^1^‐binding of the Tmp ligand.^[50]^ The solid‐state structure of dinuclear pseudo‐tetrahedral **55** was eventually disclosed in 1994, with U−P distances of the η^5^‐bound Tmp of 2.945(3) and 2.995(3) Å, and, for the η^1^‐bound Tmp of 2.996(3) Å.^[51]^ This remains the only report of a structurally authenticated U^III^ monophospholyl complex to date. The synthesis of [U(Cp*)(Tmp)(BH_4_)]_2_ by Na/Hg reduction of [U(Cp*)(Tmp)(BH_4_)_2_] was also reported in the same paper; although no structural authentication was presented for this mixed Cp*/Tmp U^III^ complex, its ^1^H and ^31^P NMR spectra (*δ*_P_: 3672 ppm, *W*_1/2_=1600 Hz; 3886 ppm, *W*_1/2_=1130 Hz) indicated similar dynamic solution behaviour to that shown by **55**. Addition of THF to [U(Cp*)(Tmp)(BH_4_)]_2_ allowed the characterisation of [U(Cp*)(Tmp)(BH_4_)(THF)] by NMR spectroscopy.

**Scheme 8:**
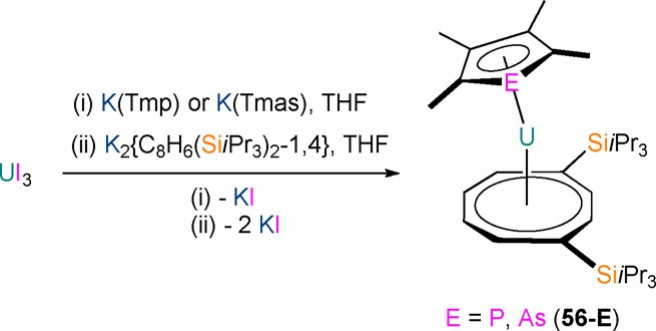
Synthesis of **56‐E** (E=P, As) from the one‐pot stoichiometric reaction of UI_3_, K_2_{C_8_H_6_(Si*i*Pr_3_)_2_‐1,4} and either K(Tmp) or K(Tmas).^[52]^

It is noteworthy that, in 2002–2003, Cendrowski‐Guillaume et al. reported that the mixed sandwich U^III^ complex [U(COT)(Tmp)(HMPA)_2_] could be synthesised by the Na/Hg reduction of the U^IV^ precursor [U(COT)(Tmp)(HMPA)_2_][BPh_4_] (see Section 6.2).[^45^, ^46^] Although this U^III^ complex was not structurally authenticated at the time, the presence of an additional coordinated molecule of HMPA over the analogous Nd^III^ complex **52** (see Section 5.3)^[46]^ was assigned by the authors, based on elemental analysis and ^1^H NMR spectroscopy data. The reaction of [U(COT)(Tmp)(HMPA)_2_] with NEt_3_HBPh_4_ gave a ^1^H NMR spectrum that was consistent with the formation of the U^IV^ complex [U(COT)(HMPA)_3_][BPh_4_]_2_ by concomitant protonolysis of Tmp and oxidation of U^III^ to U^IV^; the mechanism of this reaction was not discussed.[^45^, ^46^] In 2015, Cloke and co‐workers reported the one‐pot stoichiometric salt metathesis reactions of UI_3_, K_2_{C_8_H_6_(Si*i*Pr_3_)_2_‐1,4} and either K(Tmp) or K(Tmas) in THF to give the respective U^III^ phospholyl, [U{C_8_H_6_(Si*i*Pr_3_)_2_‐1,4}(η^5^‐Tmp)(THF)] (**56‐P**), or arsolyl complex [U{C_8_H_6_(SiMe_3_)_2_‐1,4}(η^5^‐Tmas)(THF)] (**56‐As**), following work‐up and recrystallisation (Scheme 8).^[52]^

Complexes **56‐E** were characterised by mass spectrometry, elemental analysis, single‐crystal XRD and ^1^H and ^29^Si NMR spectroscopy, and by ^31^P NMR spectroscopy for **56‐P** (*δ*_P_: 846.2, *W*_1/2_=411 Hz); no ^75^As NMR spectrum was reported for **56‐As**.^[52]^ The solid‐state structures of **56‐E** revealed similar bent metallocene‐like arrangements with an equatorial molecule of THF, η^8^‐bound {C_8_H_6_(Si*i*Pr_3_)_2_‐1,4} and η^5^‐bound Tmp/Tmas, with disorder of the Tmp ligand giving two U−P (2.776(15) and 2.9868(14) Å) and two U−Tmp_centroid_ (2.54(2) and 2.59(2) Å) distances for **56‐P**; complex **56‐As** is the only structurally authenticated An arsolyl complex to date and exhibits a U−As bond length of 3.0781(7) Å and a U−Tmas_centroid_ distance of 2.5962(4) Å. The coordinated THF in **56‐E** can be removed upon exposure of powdered samples to vacuum, and the reaction of desolvated **56‐P** with CO_2_ gave the dinuclear U^IV^ phosphacarbonate complex [{U[C_8_H_6_(SiMe_3_)_2_‐1,4](μ,κ^2^‐O_2_CPC_4_Me_4_)}_2_(μ‐O)]. The authors monitored the corresponding reaction of **56‐P** with ^13^CO_2_ by ^13^C NMR spectroscopy, and proposed a U^IV^ μ‐oxo intermediate “[{U[C_8_H_6_(SiMe_3_)_2_‐1,4](PC_4_Me_4_)}_2_(μ‐O)]” forms first by the reduction of CO_2_ to CO and oxidation of the two U^III^ centres, followed by insertion of two molecules of CO_2_ to form the observed product; analogous CO_2_ activation chemistry was observed for a homologous pyrrole complex **56‐N**.

### 6.2 An^IV^ complexes

**Scheme 9:**
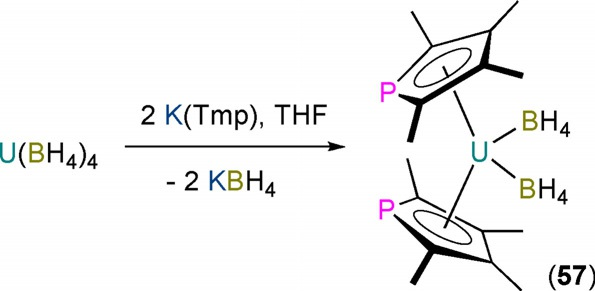
Synthesis of **57** from U(BH_4_)_4_ and 2 equivalents of K(Tmp).[^49^, ^50^]

The first structurally authenticated An^IV^ phospholyl complex, [U(η^5^‐Tmp)_2_(BH_4_)_2_] (**57**), was reported in 1990 by Baudry, Nief and co‐workers to form from the salt metathesis reaction of U(BH_4_)_4_ with two equivalents of K(Tmp) in THF (Scheme 9) or the oxidation of **55** with TlBH_4_.^[49]^ Single‐crystal XRD revealed that the U^IV^ centre of **57** exhibits a pseudo‐tetrahedral geometry in the solid state, with η^5^‐bound Tmp rings and U−P distances of 2.905(1) Å. Complex **57** was also characterised by elemental analysis, and ^1^H and ^31^P NMR spectroscopy (*δ*_P_: 960; *W*_1/2_=200 Hz);^[49]^ these data are comparable to those of a second U^IV^ monophospholyl borohydride complex that was not structurally authenticated, [U(Tmp)(BH_4_)_3_] (*δ*_P_: 923; *W*_1/2_=44 Hz), which was reported in a follow‐up paper in 1992.^[50]^ [U(Tmp)(BH_4_)_3_] was synthesised directly from U(BH_4_)_4_ and one equivalent of K(Tmp) in toluene, with ligand scrambling of this complex occurring in THF solutions to afford **57** and [U(BH_4_)_4_(THF)_2_].

**Figure 18:**
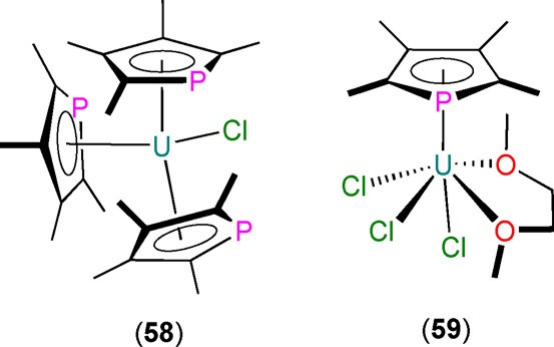
Complexes **58** ^[53]^ and **59**.^[54]^

In 1992, Ephritikhine and co‐workers reported the salt metathesis reaction of UCl_4_ with three equivalents of K(tmp) in toluene to yield [U(η^5^‐Tmp)_3_(Cl)] (**58**, Figure 18); the corresponding reaction with stoichiometric K(tmp) gave [U(Tmp)_2_(Cl)_2_].^[53]^ Several derivatives of **58** were synthesised by salt metathesis protocols with various reagents: (i) KBEt_3_H yielded the hydride [U(Tmp)_3_(H)]; (ii) MeLi gave the alkyl [U(Tmp)_3_(Me)]; and, (iii) NaO*i*Pr afforded the alkoxide [U(Tmp)_3_(O*i*Pr)]. All U^IV^ complexes in this paper were characterised by ^1^H NMR spectroscopy and elemental analysis, but only **58** was structurally authenticated, showing an approximate trigonal planar arrangement of the three η^5^‐bound phospholyls with respect to the ring centroids, identical U−P distances of 2.927(4) Å, and U⋅⋅⋅Tmp_centroid_ distances of 2.61(1) Å, with a chloride at an apical position completing the coordination sphere of the U^IV^ centre. A 1994 follow‐up full paper by Gradoz et al. outlined the synthesis of a wide range of heteroleptic U^IV^ Tmp complexes by a series of salt metathesis reactions starting from UCl_4_ or U(BH_4_)_4_ and K(Tmp) and a series of group 1 ligand transfer reagents: [U(η^5^‐Tmp)(Cl)_3_(DME)] (**59**), [U(Cp*)(Tmp)(BH_4_)_2_], [U(Tmp)(CH_2_Ph)_3_], [U(Tmp)_2_(Me)_2_], [U(Tmp)_2_(CH_2_SiMe_3_)_2_], [U(Cp*)(Tmp)(Me)_2_], [U(Cp*)(Tmp)(CH_2_SiMe_3_)_2_], [U(Tmp)_2_(OEt)_2_], [U(Tmp)_2_(O*i*Pr)_2_], [U(Tmp)_2_(O*t*Bu)_2_], [U(Tmp)_2_(Me)(Cl)], [U(Tmp)_2_(Me)(BH_4_)], [U(Tmp)_2_(CH_2_SiMe_3_)(Cl)], [U(Tmp)_2_(CH_2_SiMe_3_)(BH_4_)], [U(Cp*)(Tmp)(CH_2_SiMe_3_)(Cl)] and [U(Cp*)(Tmp)(CH_2_SiMe_3_)(BH_4_)].^[54]^ All complexes were characterised by ^1^H NMR spectroscopy and elemental analysis (except [U(Tmp)_2_(CH_2_SiMe_3_)_2_]), but a solid‐state structure was only disclosed for **59** (Figure 18). The U^IV^ centre in **59** exhibits a pseudo‐octahedral geometry with a *mer*‐configuration of Cl ligands; the two O donor atoms of DME and an η^5^‐bound Tmp ligand complete the coordination sphere, with a U−P distance of 2.926(4) Å. The synthesis of such a large number of Tmp U^IV^ complexes and analogous Cp* complexes allowed the authors to compare the steric and electronic effects of these ligands on complex spectroscopic data and redox chemistry.^[54]^

**Scheme 10:**
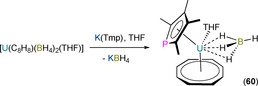
Synthesis of **60** from [U(COT)(BH_4_)_2_(THF)] with 1 equivalent of K(Tmp).^[55]^

In 2002, Cendrowski‐Guillaume et al. reported that the separate salt metathesis reactions of either [U(COT)(BH_4_)_2_(THF)] or [U(COT)(BH_4_)(THF)_2_][BPh_4_] with one equivalent of K(Tmp) yielded [U(COT)(η^5^‐Tmp)(BH_4_)(THF)] (**60**; Scheme 10).[^45^, ^55^] Treatment of **60** with a further equivalent of K(Tmp) gave the ‘ate’ complex K[U(COT)(Tmp)_2_(BH_4_)(THF)_*n*_], whilst the reaction of **60** with NaOEt furnished [U(COT)(Tmp)(OEt)]. In the same paper, the authors reported that the reaction of [U(COT)(HMPA)_3_][BPh_4_]_2_ with K(Tmp) gave [U(COT)(Tmp)(HMPA)_2_][BPh_4_], and the protonolysis reaction of this product with NEt_3_HBPh_4_ regenerated the U^IV^ starting material. The majority of these complexes were assigned by ^1^H NMR spectroscopy and elemental analysis, although a solid‐state structure was determined for **60** to reveal a U^IV^ centre coordinated by κ^3^‐BH_4_, THF, η^8^‐COT and η^5^‐Tmp, with a typical U−P distance of 2.970(8) Å.

**Figure 19:**
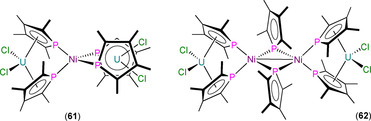
Complexes **61** and **62**.^[56]^

The only other U^IV^ monophospholyl complexes that have been structurally authenticated to date, [{U(Cl)_2_(μ;η^5^,η^1^‐Tmp)_2_}Ni(μ;η^1^,η^1^‐Tmp)]_2_ (**61**) and [U(Cl)_2_(μ:η^5^,η^1^‐Tmp)_2_Ni(μ:η^1^‐Tmp)_2_Ni(μ:η^5^,η^1^‐Tmp)_2_U(Cl)_2_] (**62**; Figure 19), were reported in 1996 by Ephritikhine and co‐workers to form by the respective Na/Hg reductions of 2:1 mixtures of either [U(Tmp)_2_(Cl)_2_] and NiCl_2_, or [U(Tmp)_2_(Cl)_2_] and [Ni(η^5^‐Tmp)(μ:η^1^‐Tmp)]_2_.^[56]^ Single‐crystal X‐ray diffraction studies of **61** and **62** revealed that the pseudo‐tetrahedral U^IV^ centres were bound by two terminal *cis*‐chlorides and two Tmp ligands in an η^5^‐binding mode in each case (**61**: range U−P: 2.823(7)–2.862(7) Å; **62**: U−P: 2.851(9) and 2.86(1) Å). The assignment of U^IV^ centres in **61** and **62** is made through analysis of Ni−P distances in the former complex being in line with Ni^0^ tetrakis‐phosphines, and a short Ni−Ni distance in the latter complex (2.546(9) Å) being consistent with the presence of a metal–metal bond and formal Ni^I^ centres; long mean U⋅⋅⋅Ni distances in these complexes (e.g., 3.38(2) Å for **61**) are not in line with 5f/3d metal–metal bonds. The ^1^H and ^31^P NMR spectra of **61** provided additional characterisation data (*δ*_P_: 199.2 ppm), whilst those of **62** were broad and could not be interpreted; crystals of **62** could not be separated easily from the NaCl by‐product, hence no additional characterisation data were obtained.

## 7 Lanthanide and Actinide Polyphospholyl Complexes

### 7.1 C_3_P_2_ and C_2_P_3_ complexes

**Scheme 11:**
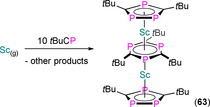
Synthesis of **63** from Sc vapour and *t*BuCP.[^57^, ^58^]

**Scheme 12:**
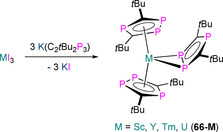
Synthesis of **66‐M** (M=Sc, Y, Tm, U) from MI_3_ and 3 equivalents of K(C_2_ *t*Bu_2_P_3_).^[63]^

A handful of examples of Ln and An polyphospholyl complexes have been reported, whilst there have been no reports to date of corresponding polyarsolyls. The first structurally characterised rare earth polyphosholyl complex, [Sc{η^5^‐C_2_ *t*Bu_2_P_3_)}_2_(μ:η^6^,η^6^‐C_3_ *t*Bu_3_P_3_)] (**63**), was reported in 1996 by Cloke, Nixon and co‐workers to form in 5–10 % isolated yield from the cyclooligomerisation reaction of *t*BuCP with Sc vapour in a 10:1 ratio (Scheme 12).^[57]^ This noteworthy triple decker complex represented the first structurally authenticated example of formal Sc^I^ centres, together with a novel instance of ligated 1,3,5‐triphosphabenzene in the solid state; f‐block complexes with Ln or An centres in formal +1 oxidation states are unknown to date. The total valence electron count of **63** is only 22 e^−^, which is also remarkably low for a triple‐decker sandwich complex. The reaction mixture that yielded **63** was further investigated by Cloke, Nixon and co‐workers, and the sandwich complex [Sc(C_3_ *t*Bu_3_P_2_)_2_] was isolated in 5–10 % crystalline yield after sublimation (Scheme 11).^[58]^ Unfortunately, this complex could not be structurally authenticated, but all characterisation data were in line with a Sc^II^ formulation. To the best of our knowledge, no Ln and An diphospholyl complexes have been structurally authenticated to date, but it is noteworthy that [Yb(C_3_ *t*Bu_3_P_2_)_2_] was made by analogous procedures and has been spectroscopically characterised.^[59]^ The diuranium complex [{U[HC(SiMe_2_NC_6_H_4_Me‐4)_3_]}_2_{μ:η^4^,η^4^‐C_2_ *t*Bu_2_P_2_}], reported by Liddle and co‐workers in 2014 to form from the reductive coupling of two molecules of *t*BuCP by a U^III^ precursor, is also worthy of mention at this point as the sole example of a Ln/An complex containing a *cyclo*‐C_2_P_2_ ring that has been structurally characterised to date;^[60]^ Liddle has recently reviewed f‐block complexes containing dianionic four‐membered aromatic rings.^[61]^

The solid‐state structure of **63** revealed that the planar bridging 1,3,5‐triphosphabenzene has elongated ring P−C bonds compared with unbound 1,3,5‐C_3_ *t*Bu_3_P_3_, together with remarkably short Sc⋅⋅⋅C_3_P_3centroid_ distances of 1.787(5) Å; this contrasts with the relatively long Sc⋅⋅⋅C_3_P_2centroid_ distances to the capping anionic C_3_ *t*Bu_3_P_2_‐1,3 rings of 2.338(6) Å (Sc−P: 2.802(2), 2.843(2) and 2.877(2) Å).^[57]^ These unusual metrical parameters indicate that significant charge transfer is present in **63**, making the formal oxidation state a moot point, but the assignment of Sc^I^ centres is a useful formalism to rationalise experimental data. Crystals of **63** exhibit a forest‐green colour, and the intense absorption in the UV/Vis spectrum of a dilute toluene solution (*λ*_max_=426 nm, *ϵ*=12 000 dm^3^ mol^−1^ cm^−1^) was assigned to a metal to ligand charge transfer band, which is typical of low oxidation state scandium; ^[57]^ a pentane solution of dark‐purple [Sc(C_3_ *t*Bu_3_P_2_)_2_] similarly exhibits a maximum absorbance at 571 nm and *ϵ*=15 000 dm^3^ mol^−1^ cm^−1^.^[58]^

Solutions of **63** were determined to be EPR silent between 298 K and 77 K,^[57]^ whilst a toluene glass of [Sc(C_3_ *t*Bu_3_P_2_)_2_] at 120 K was shown to exhibit a rich and well‐resolved EPR spectrum with hyperfine coupling of the Sc‐based unpaired electron to a 100 % abundant *I*=7/2 ^45^Sc nucleus and additional splitting by four equivalent ^31^P nuclei (100 % abundance, *I*=1/2); these features were simulated with *g*_⊥_=2.0098, *g*_∥_=1.9273, *A*_⊥_(^45^Sc)=29.9 G, *A*_∥_(^45^Sc)=52.9 G and *A*(^31^P)=7.2 G.^[58]^ A solution of **63** was additional probed by Evans method magnetic susceptibility, where the value at 295 K (3.98 μ_B_) is lower than the predicted value of 4.47 μ_B_ for four unpaired electrons arising from two isolated Sc^I^ centres.^[57]^ In contrast, the magnetic susceptibility measured at room temperature for a toluene solution of [Sc(C_3_ *t*Bu_3_P_2_)_2_] (1.70 μ_B_) is fully in accord with the expected value of 1.73 μ_B_ for a 3d^1^ system with no orbital contribution, and a more clear‐cut Sc^II^ centre; however, the stability of this complex was attributed to the capability of the diphospholyl ligands to accept electron density from the metal.^[58]^

**Figure 20:**
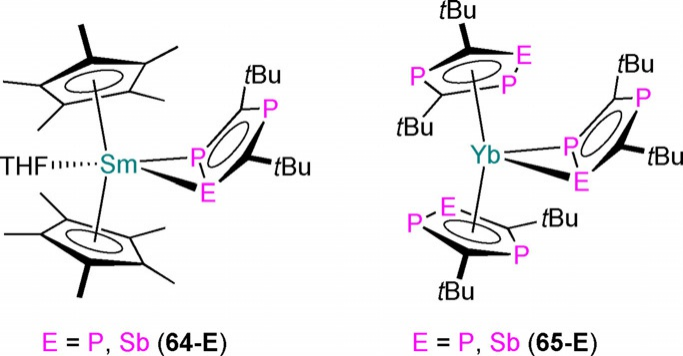
Complexes **64‐E** and **65‐E** (E=P, Sb).^[62]^

In 2000, Deacon et al. reported the syntheses of co‐crystallised [Sm(Cp*)_2_(η^2^‐C_2_ *t*Bu_2_P_2_E)(THF)] (**64‐E**, E=P, Sb; Figure 20), in 10 % yield from the SET reaction of [Sm(Cp*)_2_(THF)_2_] with [Tl(C_2_ *t*Bu_2_P_2_E)], where the Sb/P ratio of E in the Tl^I^ precursor was approximately 4:1.^[62]^ In the same paper, a mixture of [Tl(C_2_ *t*Bu_2_P_2_E)] and Yb metal in THF was sonicated for 48 h, and upon work‐up co‐crystals of [Li(THF)_4_][Yb(η^5^‐C_2_ *t*Bu_2_P_2_E)_2_(η^2^‐C_2_ *t*Bu_2_P_2_E)] (**65‐E**, E=P, Sb; Figure 20) were isolated, with the Tl^I^ precursor presumably contaminated with a significant amount of Li‐containing compounds.^[62]^ The authors made valiant efforts to determine the Sb/P ratios of E in **64‐E** and **65‐E** by ^1^H and ^31^P NMR spectroscopy, and found that for the former mixture P was in excess, whereas for the latter the Sb/P ratio was 2:1. This indicates that if pure [Tl(C_2_ *t*Bu_2_P_3_‐1,2,4)] could be obtained then it may react with [Sm(Cp*)_2_(THF)_2_] to give **64‐P** cleanly, but there are more variables to explore for the synthesis of pure **65‐P** in the future.

Recrystallisation of **64‐E** gave several crystals of antimony‐free **64‐P**, which were analysed by single‐crystal XRD, whereas the SC‐XRD dataset for **65‐E** showed the presence of both Sb and P, as well as other products containing C_2_ *t*Bu_2_P_3_ rings.^[62]^ The Sm^III^ centre in **64‐P** is coordinated by two η^5^‐Cp* ligands, a molecule of THF, and the 1,2,4‐C_2_ *t*Bu_2_P_3_ ring in an η^2^‐fashion, with relatively long Sm−P distances of 3.135(2) and 3.164(2) Å attributed to steric buttressing. The angle between Sm, the P−P bond mid‐point and the mean plane of the 1,2,4‐C_2_ *t*Bu_2_P_3_ ring in **64‐P** is 143.8°, which contrasts with the analogous approximately 180° angle shown by similar η^2^‐bound pyrazolyl complexes in the same paper such as [Yb(Cp*)_2_{C_3_HPh_2_N_2_‐3,5}]; however, although noteworthy, the differing coordination spheres of these complexes precludes a meaningful comparison. The Yb^II^ centre in the anion of **65‐E** exhibits two η^5^‐ and one η^2^‐bound C_2_ *t*Bu_2_P_2_E ring, with the major Sb‐containing component of the η^2^‐bound ring exhibiting an angle of 112.8° between the Yb‐(Sb‐P_mid‐point_) vector and the ring mean plane. The authors attributed this observation to inter‐ligand steric repulsion preventing η^5^‐coordination of the third ring; crystallographic disorder prevented the extraction of reliable Yb−Sb (3.24(3) Å) and Yb−P (3.09(2) Å) distances from these data.^[62]^

In 2003, Cloke, Green and Nixon communicated the synthesis of the Sc^III^ complex [Sc(η^5^‐C_2_ *t*Bu_2_P_3_)_2_(η^2^‐C_2_ *t*Bu_2_P_3_)] (**66‐Sc**), by the reaction of ScI_3_ with three equivalents of K(C_2_ *t*Bu_2_P_3_) in toluene under reflux (Scheme 12).^[63]^ In a follow‐up full paper in 2008, Clentsmith et al. reported the analogous synthesis and solid‐state structures of the homologous Ln^III^ and An^III^ complexes **66‐M** for M=Y, Tm and U (Scheme 12).^[64]^ The metrical parameters in the solid‐state structures of **66‐M** vary according to M^III^ ionic radii but all exhibited two η^5^‐ and one η^2^‐bound triphospholyl ligands: M−P distances to the former are 2.773(3)–2.813(3) Å (**66‐Sc**), 2.928(3)–3.059(3) Å (**66‐Y**), 2.896(2)–3.052(3) Å (**66‐Tm**) and 2.998(2)–3.114(2) Å (**66‐U**); M−P distances to the latter are 2.762(3) and 2.796(3) Å (**66‐Sc**), 2.884(2) and 2.912(2) Å (**66‐Y**), 2.853(2) and 2.891(2) Å (**66‐Tm**) and 2.968(2) and 2.995(2) Å (**66‐U**).^[63,63]^ The variable‐temperature ^31^P NMR spectra of **66‐Sc** showed an approximate (AX_2_)_3_ pattern at all temperatures investigated (although a simulation of an AA'A’’X_2_X’_2_X’’_2_ system showed there is slight deviation owing to inter‐ring coupling), suggesting that a more symmetrical arrangement of ligands exists in solution for this complex, with signals at 296.5 (A) and 265.0 (X) ppm, and coupling constants of *J*_A,X_=−50.7 Hz, *J*_A’,X_=+21.5 Hz, *J*_X,X’_=±5.0 Hz and *J*_A,A’_=0 Hz). The ^31^P NMR spectra for **66‐Y** were not as well‐resolved (*δ*_P_: 289.9 (br, 1 P), 263.9 (br, 2 P) ppm), but at 70 °C the high field signal resolved to a doublet with a splitting of 49.3 Hz, typical of a ^2^ *J*_PP_ coupling constant; the authors attributed the smaller inter‐ring coupling in this system to the rings being further apart.^[64]^ The signals in the ^31^P NMR spectra of paramagnetic **66‐Tm** and **66‐U** were broader, with the former indistinguishable from the baseline and a single observable signal for the latter at 691.5 ppm (*W*_1/2_=200 Hz).

**Scheme 13:**
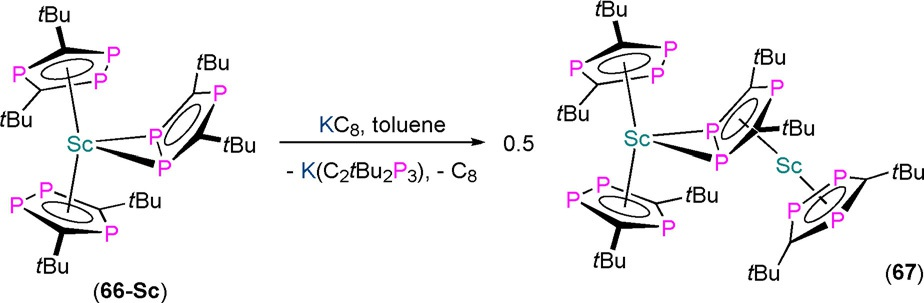
Reduction of **66‐Sc** with KC_8_ to give **67**.^[63]^

Reduction of **66‐Sc** with KC_8_ in toluene at −78 °C gave a dark‐blue solution with elimination of K(C_2_ *t*Bu_2_P_3_) and graphite, and following work‐up and sublimation at 170–180 °C and 1×10^−5^ bar, crystals of dinuclear [(η^5^‐C_2_ *t*Bu_2_P_3_)_2_Sc(μ:η^2^,η^5^‐C_2_ *t*Bu_2_P_3_)Sc(η^5^‐C_2_ *t*Bu_2_P_3_)] (**67**) were obtained (Scheme 13).^[63]^ The coordination spheres of the two Sc centres in **67** differ, with one almost identical to that seen in **66‐Sc**, with a range of Sc−P distances (2.7504(13)–2.942(2) Å) and Sc⋅⋅⋅C_2_P_3centroid_ distances of 2.322(4) and 2.360(4) Å to the η^5^‐bound rings. The second Sc exhibits a sandwich motif (range Sc−P: 2.5627(14)–2.842(2) Å), with short Sc⋅⋅⋅C_2_P_3centroid_ distances of 2.046(4) Å to the bridging ring and 2.253(4) Å to the terminal ring. This asymmetry led the authors to propose that in the solid state, Sc^I^ and Sc^III^ formalisms can be assigned to describe the electronic structure of **67** rather than two Sc^II^ centres; this is in accord with DFT calculations and powdered samples of **67** being diamagnetic by SQUID magnetometry, which led to the authors proposing an *S=*0 closed‐shell ground state. Intriguingly, the magnetic susceptibility of **67** was determined to be 1.7 μ_B_ per Sc atom in toluene solution;^[63]^ these data are analogous to [Sc(C_3_ *t*Bu_3_P_2_)_2_] (see above),^[58]^ thus the authors proposed that in aromatic solvents a monomeric complex “[Sc(C_2_ *t*Bu_2_P_3_)_2_]” with a Sc^II^ centre in equilibrium with **67**.^[63]^ No signal was observed in the EPR spectrum of a toluene solution of **67**, which was ascribed to the dimer being more favoured than for bulkier [Sc(C_3_ *t*Bu_3_P_2_)_2_], which exhibited a rich EPR spectrum (see above).^[58]^ The strong absorption in the visible region of the electronic spectrum of a dark‐blue *n*‐heptane solution of **67** provides further evidence of the presence of a low valent Sc centre (*λ*_max_=613 nm, *ϵ*=15 000 dm^3^ mol^−1^ cm^−1^).^[63]^

**Scheme 14:**
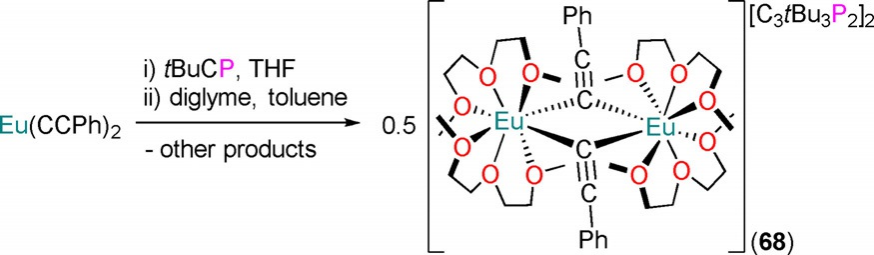
Synthesis of **68** by the sequential reaction of Eu(CCPh)_2_ with 1.2 equivalents of *t*BuCP and diglyme.^[65]^

Finally, in 2006, Deacon and co‐workers reported that the dinuclear Eu^II^ complex [{Eu(diglyme)_2_(μ‐CCPh)}_2_][C_3_ *t*Bu_3_P_2_]_2_ (**68**) formed as a minor product from the reaction of Eu(CCPh)_2_ with 1.2 equivalents of *t*BuCP in THF, followed by the addition of diglyme and toluene (Scheme 14).^[65]^ Only a small crop of yellow crystals of **68** formed from the reaction mixture, and the authors postulated that oxidative dimerisation of half of the alkynyl groups to form (CCPh)_2_ had occurred. Crystals of the cage P_5_C_5_ *t*Bu_5_ were also identified in the reaction mixture, thus the authors posited that [C_3_ *t*Bu_3_P_2_]^−^ and [C_2_ *t*Bu_2_P_3_]^−^ anions had both initially formed via phosphalkyne oligomerisation, maintaining the +2 oxidation state of Eu. Despite the low yield of **68**, this complex was characterised by single‐crystal XRD, IR spectroscopy and mass spectrometry. The dinuclear Eu^II^ dication features eight‐coordinate Eu centres with bicapped trigonal prismatic geometries; the diglyme ligands are tridentate and the two alkynyl ligands bridge to form an asymmetric Eu_2_C_2_ core. From the context of this review, the most interesting structural feature of **68** is that the two [C_2_ *t*Bu_2_P_3_]^−^ anions do not bind to the Eu^II^ centres; isolated [C_2_ *t*Bu_2_P_3_]^−^ rings had not previously been observed in the solid state. Remarkably, **68** is the only Eu phospholyl or arsolyl complex that has been structurally authenticated to date.

### 7.2 Planar *cyclo*‐P_5_ complexes

**Figure 21:**
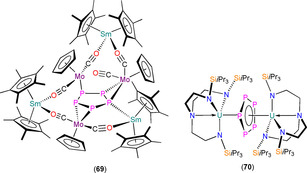
Complexes **69** and **70**.[^67^, ^68^]

Although pentaphospholyls form a unique family of complexes that are somewhat independent of the organophosphorus derivatives in the rest of this review, we include them here for completeness; there have been no reports to date of Ln or An *cyclo*‐As_5_ complexes. Metal *cyclo*‐P_5_ complexes are typically synthesised via the direct activation of white phosphorus or reactions with various P_*n*_‐transfer agents; aromatic *cyclo*‐P_5_ anions are one of a number of potential outcomes of these reactions along with a range of P_*n*_‐bound fragments, including Zintl clusters and related aromatic *cyclo*‐P_4_ dianions.^[66]^ To the best of our knowledge only one Ln and one An complex that contain planar *cyclo*‐P_5_ ligands have been structurally authenticated to date (Figure 21).[^67^, ^68^] The sole example of a structurally authenticated Ln complex containing a planar *cyclo*‐P_5_ ring, [{(Sm(Cp*)_2_}_3_(μ:η^1^,η^1^,η^2^,η^2^‐*cyclo*‐P_5_){Mo(Cp)(CO)_2_}_3_] (**69**), was reported in 2015 by Roesky and co‐workers to form as a minor product from the reduction of the P_2_ unit in [{Mo(Cp)(CO)_2_}_2_(μ:η^2^,η^2^‐P_2_)] by [Sm(Cp*)_2_(THF)_2_].^[67]^ Owing to disorder the metrical parameters from the single‐crystal X‐ray diffraction data for **69** are unreliable, but the connectivity is clear‐cut, with the planar *cyclo*‐P_5_ ring η^2^‐bound to two Mo centres and η^1^‐bound to a third, with one of the P atoms additionally η^1^‐bound to a single Sm centre (Sm−P: 2.978(11) Å). Unfortunately, owing to the low yield of **69** and co‐crystallisation with another reaction product, no additional characterisation data could be obtained.

Also in 2015, Liddle and co‐workers reported the synthesis of the dinuclear inverted sandwich uranium complex [{U[N(CH_2_CH_2_NSi*i*Pr_3_)_3_]}_2_(μ:η^5^,η^5^‐*cyclo*‐P_5_)] (**70**) from the reduction of P_4_ by the U^III^ precursor [{U[N(CH_2_CH_2_NSi*i*Pr_3_)_3_]}] in a 1:1 U/P ratio.^[68]^ The planar *cyclo*‐P_5_ ring is disordered over two positions in the solid‐state structure of **70**, which again prevents meaningful analysis of P−P distances, and the U−P distances (range 3.250(6)–3.335(6) Å) are relatively long owing to the bulky ancillary ligands. Surprisingly, the U−N distances in the ancillary ligands are in line with the presence of two identical U^IV^ centres rather than the expected mixed U(III/IV) system for a *cyclo*‐P_5_ anion. All other analytical data for **70** (NMR and UV/Vis/NIR spectroscopy, SQUID magnetometry) are also consistent with the formal presence of two U^IV^ ions and a *cyclo*‐P_5_ dianion, although such formalisms are often moot in systems with significant covalency. DFT studies of **70** showed significant δ‐donation from filled uranium 5f orbitals of appropriate symmetry to the vacant π* e_2_ orbitals of *cyclo*‐P_5_, which were again in line with significant charge transfer from uranium to the *cyclo*‐P_5_ ring. This is a consequence of both the ability of uranium to donate δ‐electron density using 5f orbitals and the superior electron accepting capability of *cyclo*‐P_5_ over Cp; the isolation of **70** versus the absence of Cp from the family of bridging *cyclo*‐C_*n*_R_*n*_ ligands (*n*=4, 6–8) in inverted sandwich An chemistry is significant.^[69]^

It is noteworthy that non‐planar *cyclo*‐P_5_ fragments were observed as part of P_10_ moieties in the Sm complexes [{Sm(C_5_Me_4_R)_2_}_2_{Fe(Cp*)_2_}_2_{μ:κ^2^,κ^2^,η^4^,η^4^‐P_10_)}] (R=Me, ^*n*^Pr), where a P−P single bond connects the two P_5_ sub‐units that are κ^2^‐bound to Sm and η^4^‐bound to Fe; these complexes were prepared in 2013 by Scheer, Roesky and co‐workers from the reactions of parent [Sm(C_5_Me_4_R)_2_(THF)_2_] with [Fe(Cp*)(P_5_)].^[70]^ Planar aromatic *cyclo*‐E_4_ dianions (E=P, As) have also been observed in f‐block chemistry, and structurally characterised examples have been shown to exhibit a range of binding modes when bridging between metal centres, with the steric effects of ancillary ligands dictating how these rings coordinate.[^67^, ^71^]

## 8 Summary and Outlook

Although group 3 and f‐block metal phospholyl and arsolyl chemistry is immature compared with cyclopentadienyls and their derivatives, some differences between the families of complexes are already evident, which provide perspectives for future exploration. Firstly, the ability of monophospholyls to stabilise low oxidation states has been demonstrated by the isolation and reactivity studies of rare examples of molecular Tm^II^ complexes;^[25]^ given the crucial role of Cp^R^ ligands in the development of low oxidation state Ln and An chemistry,^[6]^ the further exploitation of monophospholyl and ‐arsolyl ligands in synthesis and reactivity studies of analogous complexes is an obvious pathway to explore. Fine‐tuning of reduction potentials by multiple heteroatom substitution in polyphospholyl and ‐arsolyl complexes could be a useful tool in stabilising more exotic low oxidation state group 3 and f‐block complexes, as has been demonstrated in the isolation of a Sc^I^ complex.^[57]^ Polyphospholyl substituents are limited to *tert*‐butyl groups to date owing to the current reliance on *t*BuCP to generate these ligands; the development of facile synthetic routes to a wide range of polyphospholyl and ‐arsolyl ligands would be transformative in developing their f‐block chemistry to the same degree as monosubstituted analogues. Secondly, some interesting SMM properties have already been reported for Ln monophospholyl complexes;[^39^, ^48^] in view of recent reports of high blocking temperature Ln SMMs containing Cp^R^ ligands,^[7]^ it is unsurprising that Ln SMMs containing polyphospholyl ligands have already been predicted and are targets for the synthetic community.^[72]^ The optical properties of Ln phospholyl and arsolyl complexes will also vary from Cp^R^ derivatives and one can speculate that these can also be tuned by variation of the ligand field to suit specific applications.

There is considerable chemical space to explore in An phospholyl and arsolyl chemistry. Currently, there are only structurally characterised examples of such complexes for An=U; the lack of Th complexes to date is surprising given the relatively low radiological hazard of Th, the similarity of Th^IV^ and U^IV^ chemistry, and that solvated Th^IV^ starting materials are readily synthesised from commercially available precursors.^[73]^ For transuranic elements, the increasing radiological hazard across the An series limits investigations to specialist facilities,^[1b]^ but the recent extension of Cp^R^ chemistry to a structurally authenticated Am^III^ complex^[74]^ indicates that phospholyls and arsolyls can also find success for Np, Pu, Am and even beyond. Investigations into An phospholyl and arsolyl redox chemistry is also currently limited to U^III^ and U^IV^ examples, where there are a wide range of An oxidation states to explore;^[1b]^ for example, for U, Cp^R^ complexes have been structurally authenticated from the +2 to the +6 oxidation state.[^2d^, ^2e^, ^2f^, ^2g^]

There are also pathways for future exploration that are of relevance to both Ln and An phospholyl and arsolyl chemistry, which have not yet been fully exploited. Firstly, we speculate that the heteroatom lone pairs in phospholyl and arsolyl rings in η^5^‐bound complexes could be actively involved in reactivity profiles. Ln and An Cp and Cp^R^ complexes have well‐established applicability in a wide range of hydroelementation and polymerisation reactions, including catalytic processes,^[75]^ and low oxidation state Ln and An complexes of these ligands have shown rich small molecule activation chemistry.[^2d^, ^2e^, ^2f^, ^2g^] We anticipate that future investigations with analogous Ln and An phospholyl and arsolyl complexes will furnish results that complement and contrast with established Cp/Cp^R^ chemistry, with the possible involvement of P and As lone pairs in these reactions an exciting prospect. Secondly, the presence of 100 % abundant spin‐active ^31^P and ^75^As nuclei in phospholyl and arsolyl rings provides new opportunities for quantification of f‐block covalency by NMR^[76]^ and pulsed EPR^[77]^ spectroscopy. In the latter case, this has already been achieved for Th and U Cp^R^ complexes with 1.1 % abundant ^13^C nuclei, thus the presence of ^31^P or ^75^As would provide improved sensitivity, as has been shown in NMR spectroscopy covalency measurements for heteroatom‐containing ligands.^[76]^ Taking into consideration the importance of minor differences in covalency between f‐block elements to their technological applications, obtaining such data is crucial for future developments.^[78]^

To conclude, although the field of f‐block phospholyl and arsolyl chemistry is in its relative infancy it has already provided important results that juxtapose with those of derivatised cyclopentadienyl f‐block complexes. Given these past successes and the potential for wide variations in chemistry with heteroatom substitution, we realistically anticipate that other exciting results will surely follow in future investigations.

## Conflict of interest

The authors declare no conflict of interest.

## Biographical Information

*David P. Mills is a Reader at the Department of Chemistry in the University of Manchester*, *where he has spent his independent career to date focusing on non‐aqueous synthetic chemistry*, *mainly in f‐block chemistry. His research interests are centred around the synthesis and study of complexes with atypical oxidation states*, *geometries, and bonding regimes*.

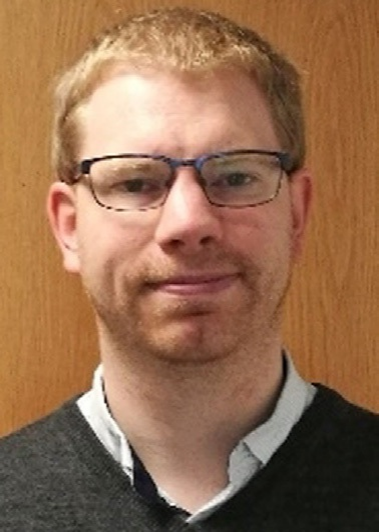

## Biographical Information

*Peter Evans received his MChem and PhD from the Newcastle University under the supervision of Dr Keith Izod*, *where he researched the stabilisation of heavier carbene analogues with bulky phosphides. He is interested the unusual reactivity of low oxidation state complexes and their synthesis*.